# *IL-1*β transgenic mouse model of inflammation driven esophageal and oral squamous cell carcinoma

**DOI:** 10.1038/s41598-023-39907-8

**Published:** 2023-08-05

**Authors:** Sureshkumar Muthupalani, Damodaran Annamalai, Yan Feng, Suresh M. Ganesan, Zhongming Ge, Mark T. Whary, Hiroshi Nakagawa, Anil K. Rustgi, Timothy C. Wang, James G. Fox

**Affiliations:** 1 Division of Comparative Medicine, Massachusetts Institute of Technology, 77 Massachusetts Avenue, 16-825C, Cambridge, MA 02139 USA; 2 StageBio, 5930 Main St, Mount Jackson, VA 22842 USA; 3 Division of Digestive and Liver Diseases and Herbert Irving Cancer Research Center, Columbia University College of Physicians and Surgeons, New York, NY 10032 USA; 4 Department of Biological Engineering, Massachusetts Institute of Technology, Cambridge, MA 02139 USA

**Keywords:** Cancer, Drug discovery, Immunology, Molecular biology, Gastroenterology, Oncology, Pathogenesis

## Abstract

Chronic inflammation is integral to the development of esophageal adenocarcinoma (EAC) and esophageal squamous cell carcinoma (ESCC), although the latter has not been associated with reflux esophagitis. The L2-*IL-1β* transgenic mice, expressing human interleukin (IL)-1β in the oral, esophageal and forestomach squamous epithelia feature chronic inflammation and a stepwise development of Barrett’s esophagus-like metaplasia, dysplasia and adenocarcinoma at the squamo-columnar junction. However, the functional consequences of IL-1β-mediated chronic inflammation in the oral and esophageal squamous epithelia remain elusive. We report for the first time that in addition to the previously described Barrett’s esophagus-like metaplasia, the L2-*IL-1β* mice also develop squamous epithelial dysplasia with progression to squamous cell carcinoma (SCC) in the esophagus and the tongue. L2-*IL-1β* showed age-dependent progression of squamous dysplasia to SCC with approximately 40% (n = 49) and 23.5% (n = 17) incidence rates for esophageal and tongue invasive SCC respectively, by 12–15 months of age. Interestingly, SCC development and progression in L2-*IL-1β* was similar in both Germ Free (GF) and Specific Pathogen Free (SPF) conditions. Immunohistochemistry revealed a T cell predominant inflammatory profile with enhanced expression of Ki67, Sox2 and the DNA double-strand break marker, *γ-*H2AX, in the dysplastic squamous epithelia of L2-*IL-1β* mice. Pro-inflammatory cytokines, immunomodulatory players, chemoattractants for inflammatory cells (T cells, neutrophils, eosinophils, and macrophages) and oxidative damage marker, *iNOS,* were significantly increased in the esophageal and tongue tissues of L2-*IL-1β* mice. Our recent findings have expanded the translational utility of the *IL-1β* mouse model to aid in further characterization of the key pathways of inflammation driven BE and EAC as well as ESCC and Oral SCC.

## Introduction

Cancers of the upper aerodigestive tract (UADT) include malignant neoplasms of the oral cavity, pharynx, larynx and esophagus^1^. Esophageal epithelial cancers rank seventh in terms of overall cancer incidence and sixth in cancer related mortality worldwide^2–4^. Esophageal epithelial cancers include esophageal squamous cell carcinoma (ESCC) and esophageal adenocarcinoma (EAC) that have significant overlap as well as distinct and unique signatures in their molecular and genomic mutational profiles^3–6^. Of these two types, ESCC is the most common subtype accounting for 90% of all cases with high prevalence rates in northern China, central Asia and southern Africa and associated with a 5-year survival rate of approximately 12–15%^2,3^. EAC is mainly associated with chronic gastro-esophageal reflex disease, Barrett’s Esophagus (BE) and obesity. ESCC on the other hand is mainly associated with tobacco use/smoking and alcohol overconsumption with other minor risk factors being consumption of hot beverages, history of thoracic radiation, nutritional deficiencies, intake of nitrosamine- rich or mycotoxin-contaminated foods and concurrent human papilloma virus (HPV)^1,3,6,7^.

Chronic Inflammation and associated alterations in the gut and /or oral microbiome are key players in altering tissue microenvironment leading to altered molecular profiles, increased cellular proliferation, DNA damage and cancer progression in the stomach, esophagus (EAC and ESCC), oral cavity and colon^8–12^. In humans, gastric-esophageal reflux disease (GERD), Barrett’s esophagus (BE), esophageal squamous dysplasia and esophageal squamous cell carcinoma (ESCC) have all been associated with a chronic enrichment of pro-inflammatory cytokines such as IL1-β, IL-6, IL-8, and TNFα^4,13–15^. Elevated tissue iNOS and NO lead to oxidative stress-related DNA double-strand breaks and genomic instability during esophageal squamous cell carcinogenesis in human patients and mouse models^11,16–19^. Animal models to study esophageal and oral squamous cell carcinomas (SCCs) are mostly based on chronic exposure to carcinogens such as 4-nitroquinoline-1-oxide (4NQO) or a combination of genetically engineered mice and chemical carcinogenesis models as well as orthotopic tumor xenograft models^5,20–23^. However, genetically engineered mouse models with spontaneous esophageal and/oral SCC development in an inflammatory setting are relatively sparse in the literature^24,25^. In the *p120 ctn* (catenin) conditional knockout mice, preneoplastic and neoplastic changes develop in the oral cavity, esophagus and squamous forestomach with grossly evident esophageal squamous tumors in approximately 70% of these mice by 9–12 months of age, and in some instances invasive cancers as early as 4 months of age. These tumors were characterized by NFκB, Akt, Stat-3 activation, increased proliferation, and upregulation of important innate immune players such as GM-CSF, M-CSF, MCP-1 and TNFα from tumor derived cells^24^. On the other hand, the *ED-L2/Klf4* mice that overexpresses the transcriptional regulator, Krüppel-like factor 4 (Klf4), under the control of an Epstein-Barr virus in the esophageal epithelium develop esophageal inflammation and slowly progress to dysplasia and invasive SCC by 2yrs of age with associated upregulation of proinflammatory cytokines, including TNF-α, CXCL5, G-CSF and IL-1α, in an NF-κB-dependent manner^25^. However, despite these two models, a well-defined inflammation driven mouse model of esophageal cancer addressing both ESCC and EAC would be invaluable to elucidate the complex role of inflammation in esophageal carcinogenesis.

The *IL-1β* transgenic mouse model (ED-L2-*IL-1β*) was initially characterized by our group as a model of Barrett’s Esophagus (BE)-like metaplasia, glandular dysplasia and adenocarcinoma (12–18 M) affecting the squamo-columnar junction of the murine stomach^26,27^. These mice selectively overexpress human IL-1β in the oral cavity (tongue), esophagus and squamous forestomach and hence also develop esophagitis and dysplasia, but ESCC development was not documented in previous publications^26,27^. As IL-1β is overexpressed in squamous cell carcinomas of the esophagus, oral cavity and nasal pharynx and its expression is linked to poor prognosis^4,6,13,14,22,28,29^, we sought to further characterize in detail the age-dependent progression of esophageal and tongue histopathological lesions in the transgenic *IL-1β* mice in different housing conditions and correlate with tissue inflammatory molecular expression profiles. Based on our findings, we now re-define the *IL-1β* transgenic mouse as a unique dual model that can be utilized to simultaneously study both inflammation-driven ESCC (plus oral SCC) and GEJ metaplasia/adenocarcinoma.

## Methods

### Experimental animals

The *IL-1β* transgenic mice (ED-*L2- IL-1β*) used in this study were embryo-rederived from the original L2- *IL-1β* mice (sourced from T.C.W) used to characterize it as a model of BE^26^. Specific pathogen free (SPF) and germ-free (GF) *IL-1β* transgenic mice, and wild type (WT) C57BL/6 mice were maintained in an Association for Assessment and Accreditation of Laboratory Animal Care (AAALAC) International accredited animal facility at Massachusetts Institute of Technology (MIT). The Committee on Animal Care (CAC), which is the Institutional Animal Care and Use Committee (IACUC) overseeing animal research at MIT, approved the animal study protocols. All methods were performed in accordance with the relevant guidelines and regulations for the care and use of laboratory animals. To maintain heterozygosity of the IL-1β transgene, *IL-1β* male mice were bred with C57BL/6 female mice or vice versa in both SPF and germ-free conditions. The progeny was genotyped for the presence of IL-1β transgene by a commercial vendor (Transneytx Inc., Cordova, TN) (Supplemental Fig. S1). Experimental mice used in this study included both males (m) and females (f) and were maintained for various ages as detailed in Supplemental Table S1.

### Necropsy, tissue collection and histopathology

All experimental mice were euthanized by CO_2_ euthanasia as per institutional approved protocols. On necropsy, for certain cohorts of SPF animals designated for molecular analysis, the esophagus was split longitudinally into two halves, one half was saved in 10% formalin for histopathology and the other half was divided into multiple small segments, snap frozen in liquid nitrogen and then stored at − 70 °C for molecular analysis. The tongue was also split longitudinally into two halves and processed in the same manner as the esophagus. For all SPF animals, tissue samples from gastric-esophageal junction (GEJ), forestomach, corpus, and antrum were collected for both molecular analysis and histology as described earlier^30^. As GF studies were conducted at the beginning of this multi-year longitudinal study, esophagus and tongue samples were collected for only histology while GEJ, forestomach, glandular stomach samples were collected for both histology and molecular analysis.

For histopathological analysis, tissues were processed by routine processing for paraffin embedding and Hematoxylin and Eosin (H&E) staining protocols. Histological slides were examined by a board certified pathologist (S.M) and graded for various histological parameters as described earlier for stomach^27,30^ with modifications for esophagus and tongue as needed from human grading schemes to reflect the squamous epithelial lining of these two sites^1^. Briefly, the tongue and esophagus were graded for inflammation, epithelial defects (erosions, ulcerations, loss of keratin), edema, hyperkeratosis, epithelial hyalinosis, squamous epithelial hyperplasia, metaplasia (squamous to columnar) and dysplasia/neoplasia on a scale of 0–4. For each animal, the various sub-categorical scores were added to generate a cumulative histopathological index score (HI) for each examined tissue. Squamous dysplasia/neoplasia was graded on the basis of established criteria as follows: 0: Negative for dysplasia, 1—Reactive/regenerative atypia or atypical hyperplasia; (indefinite for dysplasia) 2—Low grade squamous dysplasia/papilloma in which cytological atypia was confined to lower 2/3rd of the squamous lining; 3—High grade squamous dysplasia/high grade papilloma in which cytological atypia was evident in 2/3rd or more of the squamous epithelial lining; 3.5—Unequivocal lamina propria/muscularis mucosa invasion or borderline submucosal invasive squamous cell carcinoma; 4—unequivocal squamous cell carcinoma- submucosal invasion and/ or mural involvement. For grades below 3, a fractional score of 0.5 was given when the dysplastic changes were indeterminate between two categories in 1–3 small foci. The same scoring scheme was used to assess histopathological changes in the squamous forestomach of study animals.

In the glandular stomach, as the histopathological changes in *IL-1*β mice were consistent with previous descriptions of this model^26,27^ and were centered around the GEJ with extension into adjacent cardia and proximal 1/3rd of corpus, our gastric scoring criteria was modified to reflect this most relevant site of BE-type pathology and so, the stomach was scored separately for GEJ/cardia/proximal corpus, mid to distal corpus and antrum as detailed in Supplemental Methods S1 section. With respect to the glandular stomach, histological assessment in this model included scoring for inflammation, epithelial defects, oxyntic loss/atrophy, metaplasia (mucous type), hyperplasia and dysplasia on a scale of 0 to 4 as described earlier with adaptations as needed (Supplemental Material-Methods S1)^27,30^. The remainder of the gastric corpus (2/3rd) and antrum were also assessed for the various histopathological alterations with slight modifications as needed (Supplemental Methods S1, data not presented).

### Immunohistochemistry and quantitative image analysis

For routine immunohistochemistry, unstained paraffin embedded sections of the esophagus were incubated at 60 °C for 30 min, followed by de-paraffinization and rehydration prior to staining with specific antibodies for Ki67 (monoclonal, B56; BD Pharmingen, CA, USA), Sox2 (monoclonal, C70B1; Cell Signaling Technology, MA, USA), CD45/B200 (monoclonal, RA3-6B2; ThermoScientific, USA), CD3 (polyclonal, Cat.no: A0452; Dako, USA) FoxP3 (monoclonal, FJK-16S; eBiosciences, San Diego, CA, USA), Ly6G (monoclonal, 1A8; Bio X Cell Inc., Lebanon, NH, USA) and F4/80 (monoclonal, SP115; ThermoScientific, USA) with appropriate polymer based secondary antibodies as described earlier^31^. Immunohistochemical evaluation was done by either qualitative interpretative assessment or quantitative morphometric analysis in select cases (Ki67 and Sox2) using Image Pro 10 morphometric software program (Media Cybernetics Inc). For morphometric analysis, well-oriented sections of the esophagus from both WT (n = 5, 3 GF and 2 SPF) and IL-1β mice (n = 8, 4 GF and 4 SPF) at 12–15 M of age were selected and images were captured at 400X magnification. For analysis, a minimum of 500 nuclei or up to ten 400 × HPF were selected for segmentation using parameters for nuclear size for counting of positively stained and negative nuclei (for both Ki67 and Sox2) and values were expressed as number of nuclei/400X HPF and percentage of positive and negative nuclei per 400XHPF. Additionally, immunofluorescent staining for γH2AX was also performed as described earlier^31^. Briefly, the slides were incubated with mAb for γH2AX (1:200 dilution) followed by incubation with Alexa Fluor 488-conjugated anti-rabbit 52 F(ab′)2 fragment (1:750, Cell Signaling). The cell nuclei were stained using 10 μl of Prolong Gold 53 Antifade Reagent with DAPI (Cell Signaling). For morphometric analysis, the number of γH2AX positive nuclei were counted per 400X HPF and expressed as a percentage of total DAPI stained nuclei.

### Quantitative real-time and semi-quantitative PCR analysis

For measuring the relative mRNA expression of target genes, total RNA from esophagus and tongue from WT and *IL-1β* mice (n = 10–13 per group, SPF only) was extracted using Trizol reagent following manufacturer’s instructions (Invitrogen, Carlsbad, CA). Specifically, 2 µg (esophagus) or 4 µg (tongue) of the total RNA from each sample was reverse transcribed into cDNA using the High Capacity cDNA Archive kit (Life Technologies). Levels of *Il-6, Tnf-α, Inf-γ, Il-33, Il-17A, Foxp3* + and *iNos* mRNA in the esophageal and tongue tissues were measured by qPCR using commercial primers and probes (TaqMan Gene Expression assays) in the 7500 FAST real-time PCR system. The mRNA levels were normalized to the endogenous control glyceroldehyde-3-phosphate dehydrogenase mRNA (*Gapdh*) and expressed as fold change in reference to wild type littermates using the comparative threshold cycle (C_T_) method (Applied Biosystems User Bulletin No.2). Human IL-1β expression was determined by semi-quantitative PCR (Supplemental Fig. S1) using forward (5ʹ-ACCTCCAGGGACAGGATATGG-3ʹ) and reverse (5ʹ-CTCCAGCTGTAGAGTGGGCTTAT-3ʹ) primers with PCR condition 95 °C for 3 min, 40 cycles of 95 °C for 30 s, 60 °C for 30 s, 72 °C for 30 s and 72 °C for 5 min.

### Multiplex cytokine/chemokine array

For measuring cytokines and chemokines, the esophageal tissues (n = 13, WT SPF and n = 12, *IL-1β* SPF mice) were homogenized in RIPA buffer with protease inhibitors and the supernatant was collected after centrifugation at 14,000 rpm for 10 min at 4 °C. Then, the samples were normalized to contain equal amount of protein and analyzed using Luminex xMAP technology for multiplexed quantification of 32 Mouse cytokines, chemokines, and growth factors. The multiplexing analysis was performed using the Luminex™ 200 system (Luminex, Austin, TX, USA) by Eve Technologies Corp. (Calgary, Alberta). Thirty-two markers were simultaneously measured in the samples using Eve Technologies' Mouse Cytokine 32-Plex Discovery Assay® (Millipore Sigma, Burlington, Massachusetts, USA) according to the manufacturer's protocol. The 32-plex consisted of Eotaxin, G-CSF, GM-CSF, IFNγ, IL-1α, IL-1β, IL-2, IL-3, IL-4, IL-5, IL-6, IL-7, IL-9, IL-10, IL-12 (p40), IL-12 (p70), IL-13, IL-15, IL-17, IP-10, KC, LIF, LIX, MCP-1, M-CSF, MIG, MIP-1α, MIP-1β, MIP-2, RANTES, TNFα, and VEGF. Assay sensitivities of these markers range from 0.3 to 30.6 pg/mL for the 32-plex. Individual analyte sensitivity values are available in the Millipore Sigma MILLIPLEX® MAP protocol.

### Image acquisition and digital processing

H&E images from microscopic slides were acquired using Olympus BX41 microscope, Ikona 3CMOS camera (Teledyne Photometrics, Arizona, USA) and the Image Pro Plus software (Media Cybernetics, Maryland, USA). Fluorescent images were acquired using Zeiss Axioscope 2plus fluorescent microscope system with Q imaging camera (Teledyne Photometrics, Arizona, USA) and Image Pro Plus® imaging software. Gel images were obtained using GBOX CHEMI 16 GelDoc system, Syngene, Maryland, USA.

Digital image enhancements were performed using Adobe Photoshop and/or Microsoft Power Point software. Image enhancements performed included adjustments for brightness, contrast, exposure level adjustments as applied to whole images and image cropping as needed.

### Statistical analysis

Semi-quantitative histopathological scores for various categories in each tissue were analyzed by Mann–Whitney *U* test. Quantitative data values generated from tissue morphometric analysis and gene and protein expression assays were analyzed using the two-tailed Student *t* test. *P* values of ≤ 0.05 were considered significant.

### Study reporting

The study design, animal use and all experimental methods were conducted and reported in accordance with ARRIVE guidelines (https://arriveguidelines.org).

## Results

*IL-1*β mice develop inflammation-driven and age-dependent progression of squamous epithelial hyperplasia, dysplasia and squamous cell carcinoma (ESCC) in the esophagus and tongue.

**Figure 1:**
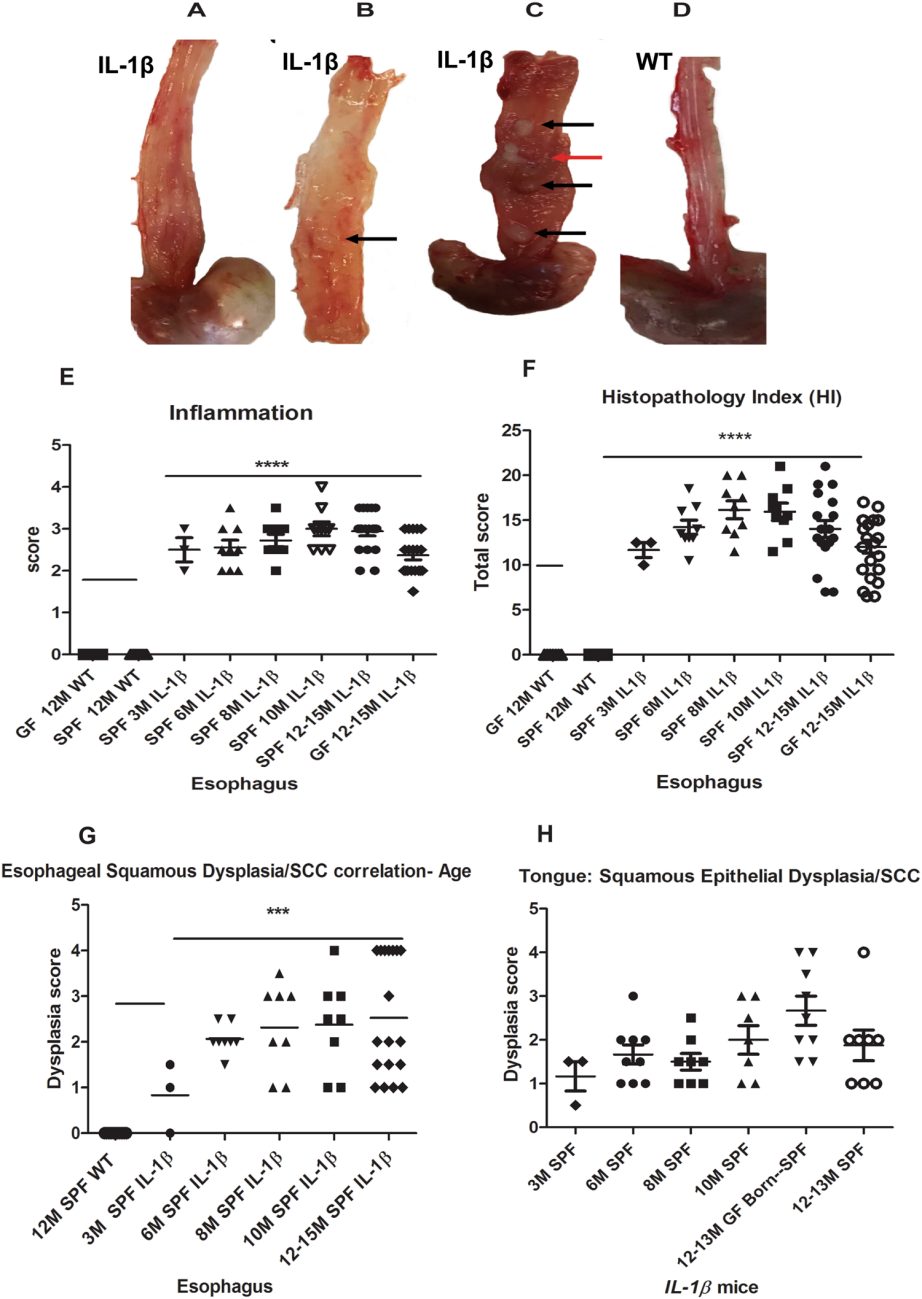
Gross pathology and age-dependent histopathological score charts in the esophagus and tongue of L2*-IL-1β* mice. (**A**–**D**) Representative gross photographs of the esophagus of *IL-1β* and WT mice. (**A**) Moderate to mid lower esophageal hypertrophy and dilatation of a 10 month (M) *IL-1β* mouse. (**B**) Focal tan-white well demarcated tumor (arrow) in mid esophagus with thickening of lower esophagus of a 12 M *IL-1β* mouse. (**C**) Diffuse thickening of esophagus with multiple tan-white well-demarcated tumors—mid to distal esophagus (black arrow) and a coalescing cavitated lesion (red arrow) in a 13 M *IL-1*β mouse. (**D**) Normal esophagus in a 12 M wild type control mouse. (**E**) Scatter dot plot of esophageal inflammation scores in different age groups, 3 M to 12–15 M in *IL1β* mice under specific pathogen free conditions (SPF). Note: WT littermate controls (SPF and germ free (GF), 12–15 M) and 12–15 M GF *IL1β* mice are also shown in the plot. (**F**) Scatter dot plot of cumulative esophageal histopathology index (HI) scores in different groups of *IL1β* mice. (**G**) Age-wise progression in esophageal squamous epithelial dysplasia in *IL1β* mice under SPF conditions (3 M to 12–15 M) compared to WT. (**H**) Scatter dot plot of *IL1β* mice showing tongue squamous epithelial dysplasia scores vs age. Dysplasia scores greater than 3 indicates invasive SCC. P values where significant are denoted by *. Individual n numbers per group/timepoint are listed in Supplemental Table S1.

At necropsy, *IL-1β* transgenic mice appeared smaller and unthrifty than age matched WT littermates. On gross examination, esophageal gross abnormalities were noted as early as 3 months in embryo re-derived L2*-IL-1*β mice over multiple generations in the form of mildly thickened mucosa/walls that progressed upon aging to more severe lesions predominantly involving the lower 2/3^rd^ of the esophagus and frequently with one or more nodular tumors (1–2 mm or more), especially in animals aged 12 months or more in both sexes (Fig. 1A–D). The tongue of *IL-1*β mice was irregularly thickened especially at 10–12 months of age (not shown). Though the gastric-esophageal junction (GEJ) and stomach were not the focus of this study as these lesions have been extensively characterized in previous studies^26,27^, we also noted similar gross abnormalities in the GEJ, cardia and proximal corpus of *IL-1*β mice (data not shown). We did not identify any tumors nor significant pathological abnormalities in the squamous stomach of *IL-1*β mice.

**Figure 2:**
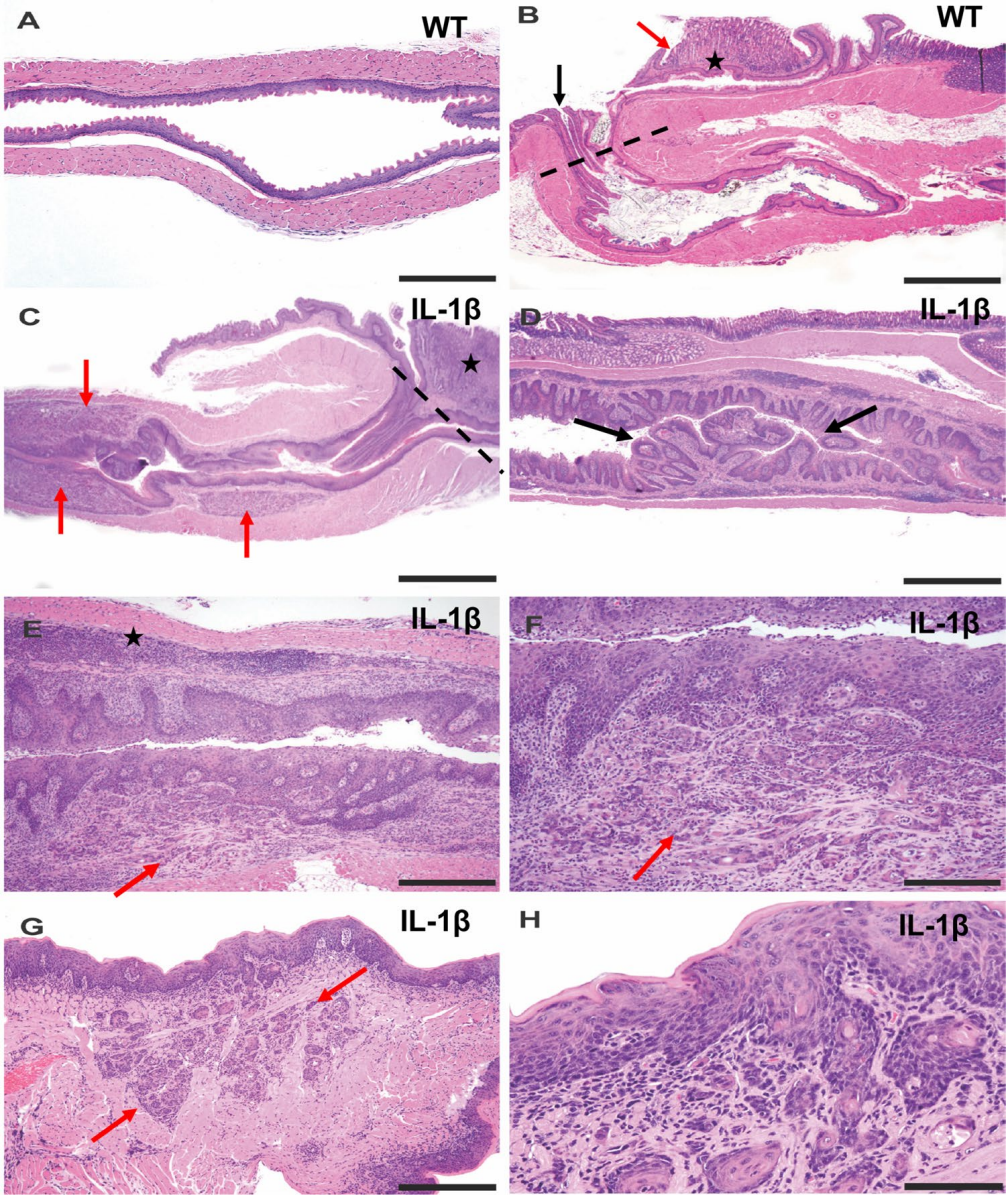
Histopathology of esophageal and tongue squamous cell carcinoma in *IL-1β* transgenic mice. (**A**) Representative hematoxylin and eosin (H&E) image of a normal esophagus in a 12 M WT mouse. (**B**) Low magnification topographical image from a 12 M WT control showing normal tubular esophagus, gastric esophageal junction (GEJ) (dashed line) transitioning into squamous stomach (black arrow) and glandular stomach (star). (**C**) Low magnification topographical image from 12 M *IL-1β* mouse showing tubular esophagus with invasive SCC foci (red arrows), GEJ transition (dashed line) and squamous stomach and cardia (star) with inflammation and metaplasia. (**D**–**F**) Representative H&E tumor images of the esophagus from a 13 M *IL-1β* mice. (**D**) Section from tumor and adjacent esophageal mucosa showing inflammation, squamous epithelial hyperplasia and papilloma (arrows). (**E**) Low magnification histological image of a grossly thickened esophagus characterized by erosion, loss of keratin layer, inflammation (star), squamous epithelial hyperplasia and high grade squamous epithelial dysplasia with focal submucosal invasive lesions consistent with squamous cell carcinoma (red arrow). (**F**) Higher magnification image of (**E**) showing submucosal downward haphazard spread of moderate to poorly differentiated neoplastic squamous cells in a fibro-inflammatory stroma (red arrow). (**G**,**H**) H&E images of tongue from a 12 M *IL-1β* mouse. (**G**) Depicts epithelial hyperplasia and invasive neoplastic squamous epithelial cell nests (arrows) deep within the tongue musculature. (**H**) Higher magnification image of (**G**) showing inflammation, hyperplastic and dysplastic squamous epithelium with invasive buds/nests. Scale bars 500 µM (panels **B**–**D**), 200 µM (panels **A**,**E**,**G**), 100 µM (Panel **F**), and 50 µM (Panel **H**).

In previous studies^26,27^ only minimal descriptions of acute and chronic esophagitis and dysplasia were noted but squamous cell carcinomas were not described. In our histopathological analysis of L2-*IL-1*β mice housed at MIT under different conditions (SPF and GF), we observed that these mice developed significant (P ≤ 0.0001) esophageal inflammation and cumulative histopathology index scores (HI) even at 3 months of age that was consistently maintained up to 15 months of age (Fig. 1E,F) compared to their WT control littermates (Fig. 2A,B). Inflammation was comprised of granulocytes (neutrophils and eosinophils) admixed with aggregates of lymphocytes, plasma cells and macrophages (Supplemental Fig. S2). In the esophagus of *IL-1*β mice, CD3 + T cells, Ly6G + cells (mature neutrophils and granulocytes) and F4/80 + macrophages were the most abundant. CD3 + T cells were present throughout the mucosa and submucosa of dysplastic and invasive SCC lesions whereas Ly6G + cells were more frequently noted within epithelial surface and beneath eroded mucosa. F4/80 + cells were more prominent within the lamina propria of the basal half of epithelium and submucosa as well within invasive foci. CD45/B220 + B cells were primarily localized within discrete submucosal lymphoid aggregates of the esophagus of *IL-1*β mice. FoxP3 + regulatory T cells were minimally increased and scattered throughout the esophageal epithelium of *IL-1*β mice while they were not noted in WT mice. Inflammatory cells were not a feature in the esophagus of WT mice (Supplemental Fig. S2) other than rare (0–1 per high power field) basal macrophages or lymphocytes that were noted in a few animals. Other associated changes included multifocal epithelial defects with ulcers/keratin loss, epithelial kerato-hyaline inclusions, multifocal to diffuse squamous epithelial hyperplasia and variable dysplastic changes in the squamous epithelium (Figs. 1G and 2C–F). Esophageal squamous epithelial hyperplasia and dysplastic changes in *IL-1*β mice were more pronounced at 10–15 months of age. The dysplastic changes ranged from reactive atypia/atypical hyperplasia to low grade dysplasia/papilloma, high grade dysplasia/papilloma and invasive squamous cell carcinoma development between 10 to 15 months of age compared to normal esophagus in wild type littermates (Figs. 1G and 2A–F). The invasive lesions consisted of moderately to well-differentiated squamous invasive nests with kerato-hyaline cytoplasmic appearance (Fig. 2E,F). The squamo-columnar junction within the actual stomach also showed hyperplastic and hyperkeratotic squamous epithelial changes but no high-grade squamous dysplasia nor SCC. Unlike the esophageal squamous epithelium, the actual squamous forestomach of *IL-1*β mice was mostly unremarkable on histological evaluation.

Similar to previous studies^26^, we also noted inflammation, mucous- type metaplasia, glandular hyperplasia and dysplastic changes within the GEJ/ gastric cardia and proximal corpus in *IL-1*β mice of both GF and SPF mice with significantly higher total pathology scores at 12–15 months but with no differences in individual categories (Supplemental Fig. S3). There was also variable milder pathological changes involving the mid to distal corpus (2/3^rd^ corpus) of *IL-1*β mice with no significant differences in pathology between GF and SPF conditions, and WT mice cohorts had stomach morphological within normal limits (data not shown). The antrum was mostly unaffected with occasional minimal to mild inflammation and hyperplasia affecting a small proportion of animals in both GF and SPF *IL-1*β mice (data not shown) but not in WT mice.

In a subset of mice, the tongue was also examined histologically and not surprisingly, the tongue of *IL-1*β mice showed mild to moderate inflammation epithelial erosions, keratin loss, squamous epithelial hyperplasia, dysplasia and progression to squamous cell carcinoma by 12 months of age (Figs. 1H, and 2G,H) compared to normal epithelium in wild type litter mates (not shown).

### Esophageal squamous dysplasia/carcinoma development in *IL-1β* transgenic mice is independent of gut microbiome

**Figure 3:**
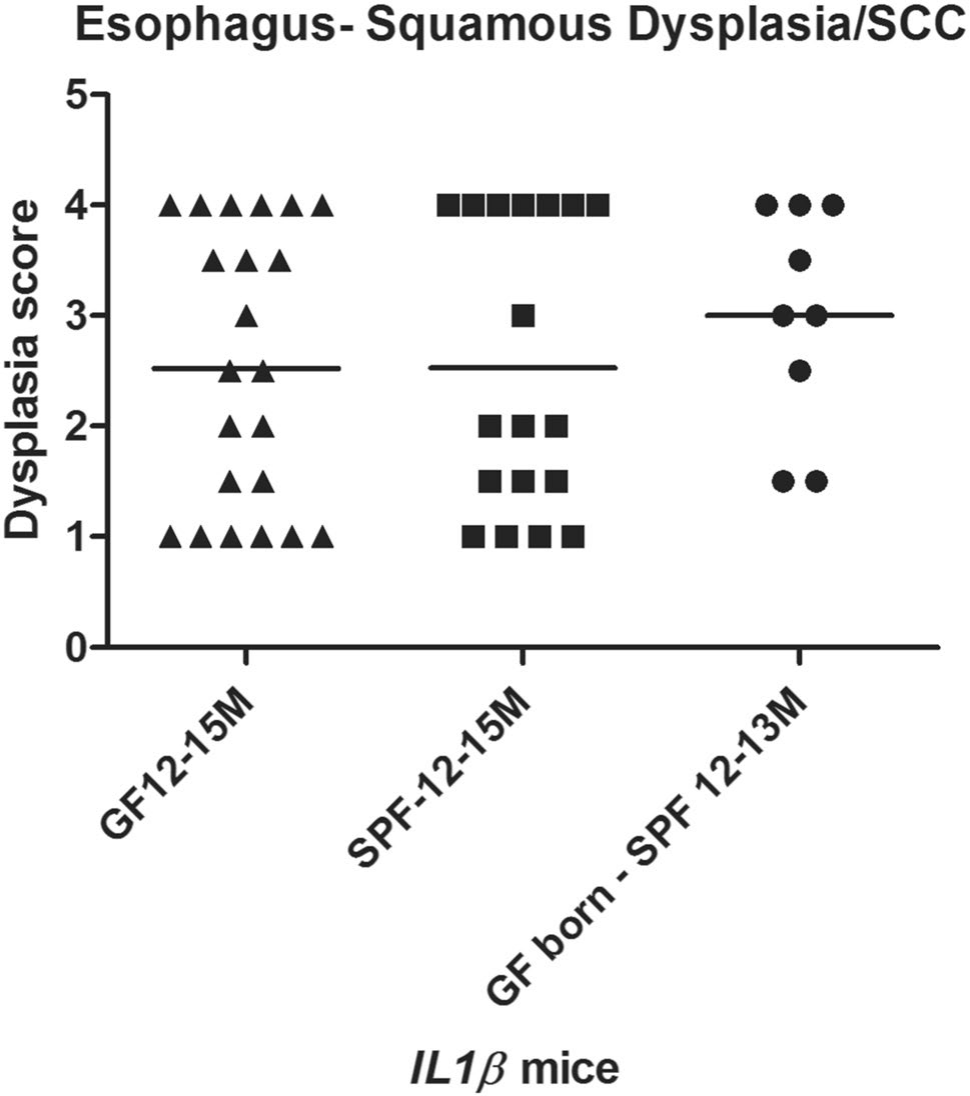
Squamous Dysplasia/ESCC incidence in *IL-1β* mice at 12–15 months in GF, SPF or mixed (GF born-SPF) conditions. Scatter plot of esophageal squamous dysplasia scores in different groups of mice. *IL-1β* mice: GF, n = 22; SPF, n = 18; GF born-SPF, n = 9.

We analyzed histopathological data from *IL-1β* transgenic mice at 12–15 months of age over multiple generations under different housing conditions, i.e., SPF, GF and GF born-transferred to SPF conditions (GF born-SPF) at 3–4 months of age to delineate microbial contribution, if any. As shown in Fig. 3 and Table 1, there was no significant difference between the three groups in terms of the overall incidence of dysplastic changes and ESCC incidence at 12–15 months of age. Interestingly, at least 40% of *IL-1β* mice are prone to develop ESCC in all three housing conditions (GF, n = 22; SPF, n = 18; and mixed GF born-SPF, n = 9) at 12-15 M and that approximately 60% (SPF and GF) to 77% (GF born-SPF) of these animals had either esophageal squamous dysplasia and/or ESCC. These changes were observed in both sexes with a trend for higher HI scores and an overall higher incidence of ESCC in females vs males (44% vs 33). Squamous non-invasive tumors and invasive ESCC’s were moderately to poorly differentiated with rare mucous type differentiation (< less than 5%) in a few cases. The consistent nature of squamous epithelial dysplasia, with a high degree of carcinoma transformation in a chronic inflammatory state of the esophagus in these *IL-1β* mice, irrespective of the presence or absence of esophageal microflora, is indicative of the inherent robust inflammatory nature of this model of ESCC. Nevertheless, though not statistically significant, there was a noticeable trend for lower scores for esophageal inflammation and cumulative HI scores in GF state as compared to SPF conditions (Fig. 3 and Table 1). Further, as shown in Fig. 1H, the tongue of *IL-1*β mice also showed low-grade dysplastic squamous epithelial changes at 3 months of age, and these lesions progressed to high-grade dysplasia by 10 months and overt SCC was found in 4/17 animals by 12 months of age under SPF conditions. The incidence of SCC in the tongue (23.5%, n = 17) was however lower than that observed for the esophagus (approx. 40%, 20 out of 49) in these SPF *IL-1*β mice. Interestingly, in the gastric-esophageal junction (GEJ)/ gastric cardia and proximal corpus of *IL-1*β mice, the cumulative histopathological scores were significantly higher under SPF conditions vs GF state (Supplemental Fig. S3). BE-like mucous metaplasia and associated dysplastic glandular changes were noted at the GEJ of *IL-1*β mice in all housing conditions (GF, GF born-SPF and SPF).

**Table 1:** Incidence of esophageal squamous epithelial dysplasia and ESCC in *IL-1β* mice.

*IL-1β* MICE (12-15 M)—Esophagus	Number of mice		
Histopathological grading criteria	GF	SPF	GF born-SPF
Normal	0	0	0
Reactive/atypical hyperplasia	8	7	2
Low grade dysplasia/papilloma	2	3	0
High grade dysplasia/papilloma	3	1	3
Invasive SCC—lamina propria	3	0	1
Invasive SCC—submucosal	6	7	3
Total number of animals	22	18	9

Dysplasia/neoplasia	% Incidence		
SCC	40%	38%	44%
Non-invasive squamous Dysplasia—low and high grade	22%	22%	33%
Dysplasia/Invasive SCC—combined	62%	60%	77%

*GF* germ free, *SPF* specific pathogen free, *SCC* squamous cell carcinoma.

### IL-1β overexpression leads to increased chemokines responsible for the recruitment of various inflammatory cells in the esophagus

**Figure 4:**
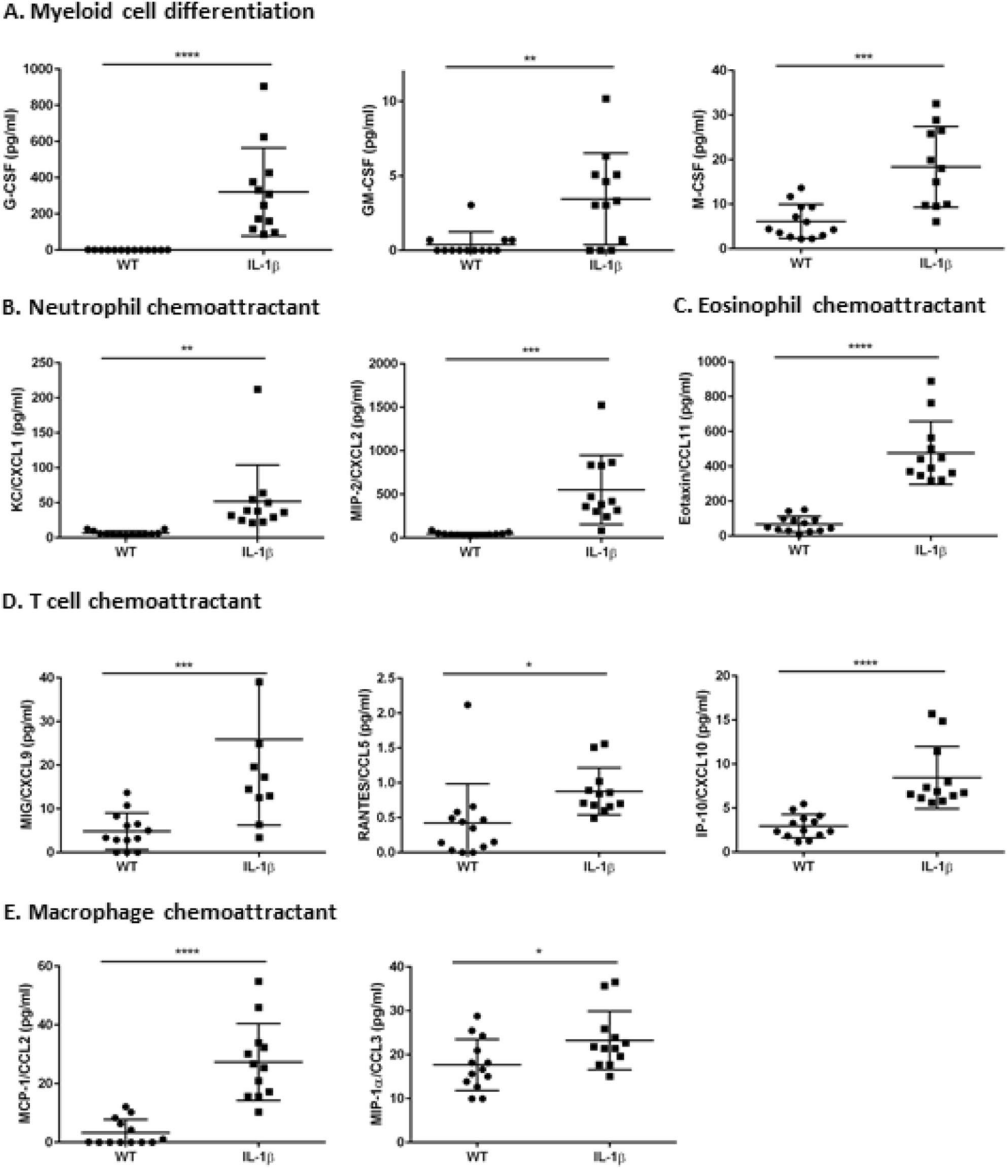
Increased expression of chemoattractants in the esophagus of *IL-1β* transgenic mice. Quantification of chemoattractants for various inflammatory cells were done in the esophageal tissues of 12 M old WT and *IL-1β* SPF mice. Chemokines for (**A**) myeloid cell differentiation (G-CSF, GM-CSF and M-CSF), (**B**) Neutrophils (Eotaxin and MIP-2), (**C**) Eosinophils (KC and MIP-2), (**D**) T cell (MIG, RANTES and IP-10), (**E**) Macrophages (MCP-1 and MIP-1α) were determined in the esophageal tissue. n = 13, WT- SPF and n = 12, *IL-1β*- SPF. Unpaired two tailed t test, *p ≤ 0.05, **p ≤ 0.01, ***p ≤ 0.001, ****p ≤ 0.0001.

Due to inherent inflammatory nature of the transgenic *IL-1β* mouse model^26,27,32^, we sought to determine expression of various chemokines required for recruitment of inflammatory cells into the esophagus of these mice. As shown in the Fig. 4, several chemokines required for recruitment and differentiation of myeloid cells M-CSF, GM-CSF and G-CSF were significantly increased in the esophagus of *IL-1β* transgenic mice. This suggests a favorable microenvironment for homing of myeloid cells in the esophagus of *IL-1β* mice as demonstrated earlier by immunohistochemistry for various inflammatory cells (Figure S2). This is consistent with the previous finding of increased myeloid derived suppressor cells at the GEJ junction in this model^26,27^. In addition, as shown in Fig. 4B–E, potent chemoattractants for recruitment of neutrophils (KC/CXCL1 and MIP-2/CXCL2), eosinophils (Eotaxin/CCL11), macrophages (MCP-1/CCL2 and MIP-1α/CCL3) and T cells (MIG/CXCL9, RANTES/CCL5 and IP-10/CXCL10) were also significantly increased in the esophageal microenvironment. Collectively, the protein expression data in correlation with tissue inflammatory cellular profiles implicate a key role for inflammatory microenvironment in the progression of esophageal squamous dysplasia/carcinoma in this model.

### IL-1β overexpression establishes pro-inflammatory microenvironment in the esophagus

**Figure 5:**
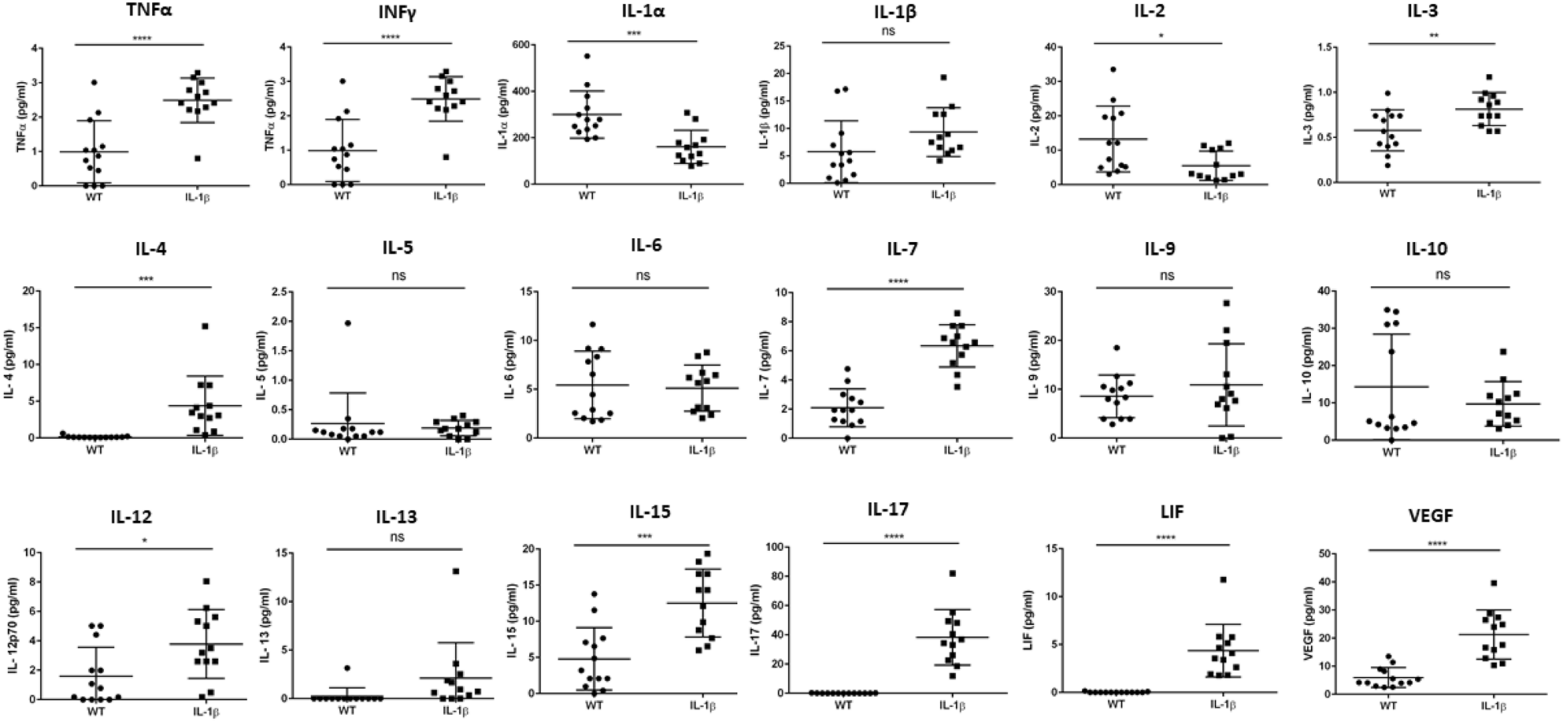
IL-1β overexpression alters cytokine landscape in the esophagus. Quantification of various cytokines in the esophagus of 12 M old WT and *IL-1β* SPF mice. TNFα, IL-3, IL-4, IL-7, IL-12, IL-15, IL-17, LIF and VEGF were significantly increased in the esophagus of IL-1β transgenic mice. n = 13, WT-SPF and n = 12, *IL-1β*-SPF. Unpaired two tailed t test, *p ≤ 0.05, **p ≤ 0.01, ***p ≤ 0.001, ****p ≤ 0.0001.

While IL-1β overexpression induces the specific downstream signaling pathways (IL-6 and TNFα), the activation of these signaling pathways induce myriad of changes by inducing potent inflammatory reactions by altering cytokine landscape in the tissue microenvironment^6,26,27^. Hence, we sought to determine the expression of various cytokines in the esophageal microenvironment of *IL-1β* transgenic mice. Initially, we sought to assess mRNA levels of key inflammatory cytokines in the tongue and esophagus during squamous dysplasia/ESCC progression, especially the downstream players of the IL-1β signaling cascade. As shown in Supplemental Fig. S4, *Il-6 and Tnfα* were significantly upregulated in the esophagus and tongue of both 6 month and 12-month-old *IL-1β* transgenic mice compared to wild type littermates. In contrast, *Infγ* expression was not affected at 6 and 12 months in both the esophageal and tongue tissues of *IL-1β* mice. To further understand the inflammatory microenvironment in the esophagus, we performed multiplex cytokine analysis. As shown in Fig. 5, TNFα, a potent cytokine linked to the development of ESCC cancer, was significantly increased in the esophagus of *IL-1β* mice. Similarly, other cytokines strongly involved in the differentiation and maintenance of T and B cells such as IL-3, IL-4, IL-7, IL-12, IL-13, IL-15 and LIF are significantly increased in the esophagus of *IL-1β* transgenic mice compared to WT mice, suggesting a strong T and B cell mediated immune environment in the esophagus of these mice. While proinflammatory cytokines, TNFα and IL-6, are significantly over expressed at mRNA level in the esophagus of *IL-1β* transgenic mice, surprisingly, only TNFα was significantly increased in our multiplex cytokine array and IL-6 levels were unremarkable. This discrepancy was attributed to the detection sensitivity of the protein expression assay. Nevertheless, the role of IL-6 in the ESCC development in our model cannot be excluded. Importantly, while IL-17 was highly expressed in the esophagus of *IL-1β* mice, it is undetectable in most WT mice. Further, IL-2 which is a potent inhibitor of TH17 cell differentiation is significantly decreased in the esophagus of *IL-1β* mice. Collectively, this suggests a unique and significant role played by IL-17 and Th17 polarized cells in the development of esophageal squamous dysplasia/carcinoma in *IL-1β* transgenic mice. In addition to changes in various cytokines which regulate diverse cell populations, VEGF a potent angiogenic factor is also significantly increased in the *IL-1β* mice. Overall, the cytokine expression analysis demonstrates a strong proinflammatory landscape in the esophagus which potentially favors the progression of ESCC.

### *IL-1β* transgenic mice develop an immunosuppressive microenvironment in the esophagus through enhanced expression of *Foxp3*+*, Il-17A* and *Il-33*

Emerging evidence in several types of tumors demonstrate that tumor cells evade immune surveillance by immunosuppressive microenvironment within tumor tissues^33–35^. In a previous study, targeted overexpression of IL-1β in gastric parietal cells induced gastric adenocarcinoma by recruiting immunosuppressive MDSCs into the tumor microenvironment^32^. Interestingly, key factors known to induce immune evasion in tumor microenvironment, namely Foxp3 + (marker for T regulatory cells-Treg) (Supplemental Figs. S2, S5) and Il-17A (Fig. 5 and Supplemental Fig. S5) were significantly increased in the esophagus of IL-1β transgenic mice compared to wild type littermates. Tregs that were recruited and persistently present in the esophagus likely contributed to immunomodulation and promotion of squamous dysplasia/ESCC development. IL-17A along with Tregs has recently been reported to play a significant role in the progression and prognosis of ESCC in human patients^35–37^. IL-33 is another important cytokine which is linked to the development of immune suppression, and its upregulation has been associated with poor clinicopathological outcomes in ESCC patients^38^. Additionally, *Il-33* mRNA was also significantly over expressed at 6 months and 12 months in the esophagus and tongue of *IL-1β* mice (p ≤ 0.001) compared to WT littermates (Supplemental Fig. S5). Collectively, our results demonstrate upregulation of key immunosuppressive factors Foxp3 + regulatory cells, IL-17A and IL-33, all of which likely sustain an immunosuppressive tissue microenvironment during the progression of squamous cell dysplasia/carcinoma in the esophagus and tongue of *IL-1β* mice.

### Esophageal squamous dysplasia/ SCC in *IL-1*β transgenic mice is associated with increased epithelial nuclear Ki67 and Sox2 expression

**Figure 6:**
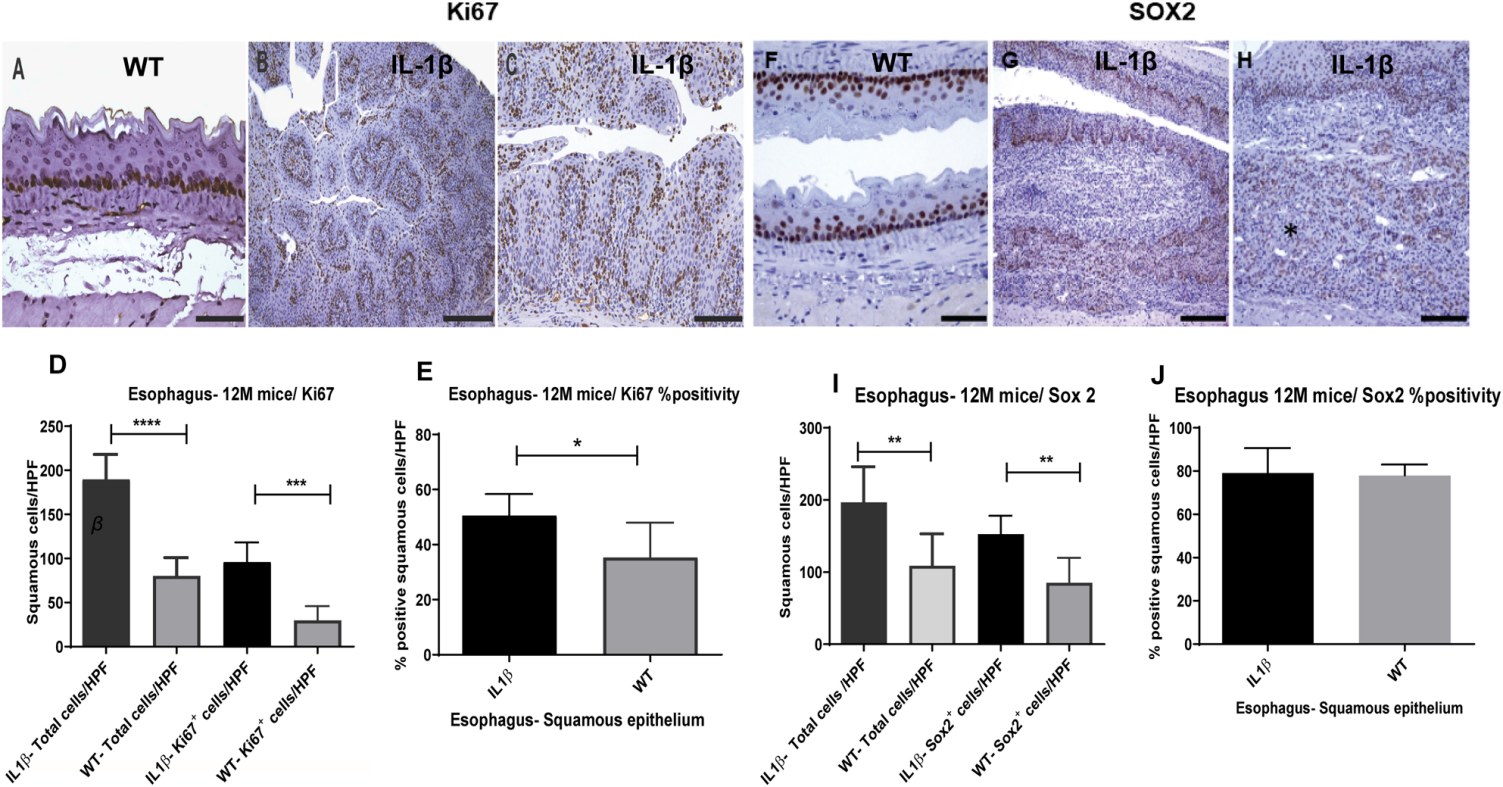
Esophageal squamous epithelial lesions in *IL-1β* mice correlate with increased Ki67 + ve cells and aberrant SOX2 immunolocalization. Representative immunohistochemical images of the esophagus of WT (**A**) and *IL-1β* mice (**B**,**C**) at 12–15 months of age: (**A**) basal Ki67 positivity of the normal thin esophageal epithelium in a representative WT mouse. (**B**,**C**) The low and high magnification images from the esophagus of *IL-1β* mouse with strong Ki67 positivity in a highly proliferative epithelium. (**D**,**E**) Depict bar charts of morphometric analysis of Ki67 positive nuclei in the squamous esophagus. (**D**) The average numbers of Ki67 positive and negative cells /40X HPF (5–6 fields/animal) of 12–15 month WT and *IL-1β* mice. (**E**) Represents % Ki67 nuclear positivity. (**F**–**H**) Representative SOX2 immunostained images of the esophagus of WT and *IL-1β* mice at 12–15 months of age. (**F**) Strong SOX2 positivity is noted in basal and parabasal squamous cells in the esophagus of a normal WT esophagus. (**G**,**H**) Low and high magnification image of the esophagus from an *IL-1β* mouse with invasive SCC showing SOX2 nuclear positivity within the dysplastic epithelium and submucosal SCC (*). (**I**,**J**) Depict the bar chart counts of morphometric analysis of SOX2 positive nuclei in the squamous esophagus. I, shows the average numbers of SOX2 positive and negative cells /40X HPF (5–6 fields/animal) of 12–15 month WT and *IL-1β* mice. (**J**) Shows % SOX2 nuclear positivity. WT (n = 5: 3 GF and 2 SPF) and IL-1β mice (n = 8: 4GF and 4SPF). Unpaired, two tailed t test. *p ≤ 0.05, **p ≤ 0.01, ***p ≤ 0.001, ****p ≤ 0.0001. Scale bars 50 µM (Panels **A** and **F**), 100 µM (Panels **C** and **H**), and 200 µM (Panels **B** and **G**).

As shown in Fig. 6A–E, the hyperplastic esophageal squamous epithelium of *IL-1β* mice correlated with significantly higher epithelial nuclear counts (both Ki67 positive and negative nuclei) per 40X high power objective fields (400X magnification) (P ≤ 0.0001) vs wild type littermates (mean total cells = 190 vs 80; mean Ki67 positive cells = 96 vs 30) at 12–15 months of age. On a normalized basis, approximately 50% of squamous epithelial cells in the *IL-1*β esophageal mucosa were Ki67 (proliferation marker) positive vs 35% positivity for the WT control epithelium (P = 0.0202) (Fig. 6D,E).

SRY (sex determining region Y)-box 2, also known as SOX2, belongs to a large family of SRY-related HMG box transcription factors and is critical for maintaining pluripotency of undifferentiated embryonic stem cells, thus imparting self-renewal and tissue regeneration properties to the immature basal cells of the squamous epithelium^39^. As SOX2 expression is increased in squamous epithelial dysplasia/ SCC of tissues including esophagus^13,28,39,40^, we evaluated esophageal SOX2 expression by immunohistochemistry in a subset of mice used in this study. As shown in Fig. 6F–H strong SOX2 nuclear positivity noted in most basal cells in both *IL-1*β and WT esophageal squamous epithelium. SOX2 positivity was also a prominent feature in the highly proliferative esophageal squamous epithelium of *IL-1*β transgenic mice and within dysplastic/invasive foci but at a lower intensity than basal epithelial cells (Fig. 6G,H), with a significant increase (P ≤ 0.0074) in the absolute numbers of SOX2 positive nuclei/HPF compared to the esophagus of WT mice (mean, 152 vs 85) (Fig. 6I). However, on a normalized basis for percentage cell positivity, SOX2 positivity percentages were similar between *IL-1*β and WT groups (approximately 78–79%) as SOX2 positive basal cells comprised a considerable proportion of normal esophageal epithelium (Fig. 6I,J).

### Esophageal dysplasia/SCC carcinoma in *IL-1*β mice is associated with increased *iNos* and double stranded DNA damage marker (γ-H2AX) expression

**Figure 7:**
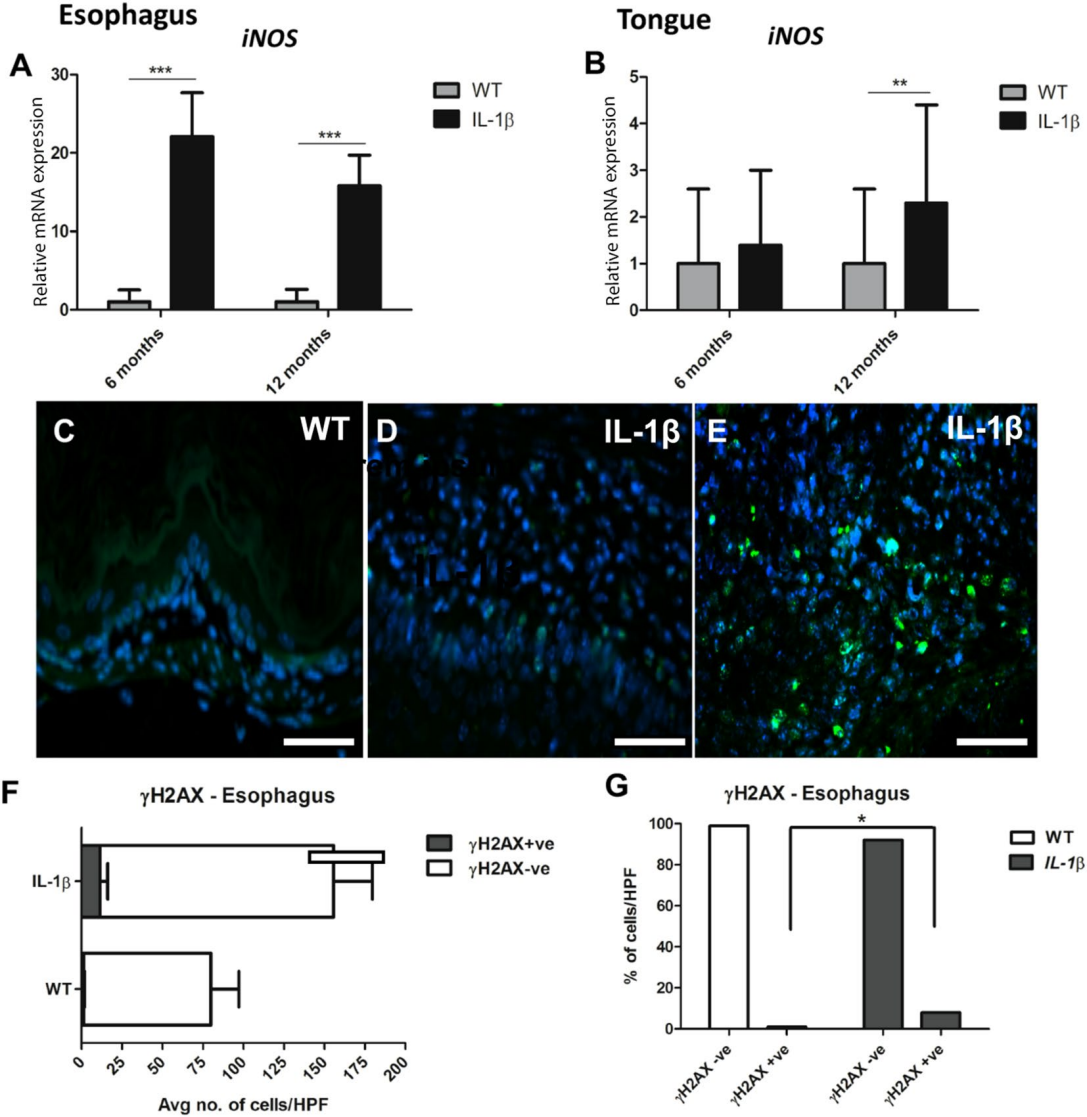
*iNos* overexpression and increased DNA double stranded breaks (γH2AX immunofluorescent staining) in *IL-1*β mice. (**A**,**B**) Upregulation of *iNos* in esophagus (**A**) and tongue (**B**) of *IL-1β* mice. Relative mRNA level of *iNos* were normalized to the expression of housekeeping gene *Gapdh*. The *y*-axes represent the mean fold changes (± standard deviation) of the mRNA levels in reference to wild type littermates. n = 10–13 per group (SPF only). (**C**–**G**) Representative γH2AX immunofluorescent images from the esophagus of WT (**C**) and *IL1β* mice (**D**,**E**), n = 5 per group- WT (GF and SPF combined) and *IL-1β* (GF and SPF combined). (**C**) Basal levels of nuclear γH2AX expression (DNA double stranded breaks marker) in normal squamous epithelium. (**D**) increased granular nuclear γH2AX positive staining within hyperplastic squamous epithelium. (**E**) Increased γH2AX nuclear positivity within esophageal squamous tumor. (**F**,**G**) Depict bar charts of the average number of γH2AX + cells/HPF and their relative percentages, respectively. WT (n = 5: 3 GF and 2 SPF) and IL-1β mice (n = 8: 4GF and 4SPF). Scale bars 100 µM (Panels **C**–**E**).

IL-1β is an important driver of inflammation that results in inducible nitric oxide synthase (iNOS) overexpression from macrophages and other cell types including epithelial cells in various tissue inflammatory states including esophageal and oral SCC’s^16,41^. As shown in Fig. 7A, iNos was significantly upregulated (p ≤ 0.001) in the esophagus of IL-1 β mice at both 6 and 12 months compared to wild type littermates. Similarly, iNos was highly expressed at 12 months (p ≤ 0.01) in the tongue of IL-1β mice (Fig. 7B). iNOS activity is strongly associated with the generation of reactive nitrogen species causing DNA damage (DNA double-strand breaks) and development of genomic instability in tumors of humans and rodent models^11,17–19^. Hence, we performed immunostaining for γ-H2AX, an established marker for detecting DNA double-strand breaks^11^. We observed significant increases in the absolute numbers of γ-H2AX + ve nuclei per 40XHPF in the esophageal epithelium of IL-1β mice compared to WT mice (12 + ve vs 1 + ve respectively; P < 0.05) (Fig. 7C–G). On a percentage basis, approximately 8% of squamous epithelial cells in the esophagus of IL-1β mice were positive for γ-H2AX as compared to less than 1% basal levels in WT mice.

## Discussion

Interleukin-1 beta (IL-1β) is a critical activator of chronic inflammation and is produced by various cells including activated macrophages, myeloid-derived suppressor cells (MDSC’s), dendritic cells and neutrophils, fibroblasts and cancer cells^42–44^. IL-1β acts as a cancer promoter with roles in cell proliferation and invasion, neo-angiogenesis, and recruitment of tumor infiltrating immune cells^42,44,45^. Other members of the IL-1 family such as IL-1α and IL-33 function as cytokine activators and increased together with IL-1β, whereas another cytokine, IL-1 receptor antagonist (IL-1RA) serves as an inhibitory cytokine^43,44^. IL-1β expression level is correlated as a predictive biomarker for oral and esophageal SCC’s^28^, and thus inhibition of IL-1β or its receptor agonists is a promising cancer therapeutic strategy^46^.

Transgenic ED L2-IL-1β mice that overexpress IL-1β is an established model of inflammation-driven Barrett’s like metaplasia and GEJ tumors. In this model, bile acids (deoxycholic acid), chemical carcinogens (e.g., MNU), high fat diet and gut microbiome were shown to promote metaplasia and tumor development^26,27^. IL-1β overexpression induced epithelial proliferation in the esophagus and stomach with activation of downstream inflammatory signaling chiefly via the IL-6/STAT3 and1L-8/CXCL1 chemokine pathways^26,27^. We now re-define the ED L2-IL-1β mice as a dual model of inflammation driven esophageal/oral SCC and BE/GEJ junctional adenocarcinoma. Our data from IL-1β mice has shown that esophageal squamous dysplasia/SCC development was independent of microbial status. This was also associated with increased Ki67 and SOX2 expression as well as γ-H2AX, a DNA double-strand break marker and increased tissue iNOS levels, indicative of inherent oxidative injury promoting mechanisms during IL-1β induced chronic inflammation. IL-1β overexpression in the esophagus and tongue was accompanied by robust recruitment of inflammatory cells and expression of proinflammatory cytokines suggestive of complex tissue microenvironmental dynamics during the progression of oral and esophageal squamous dysplasia/SCC.

**Figure 8:**
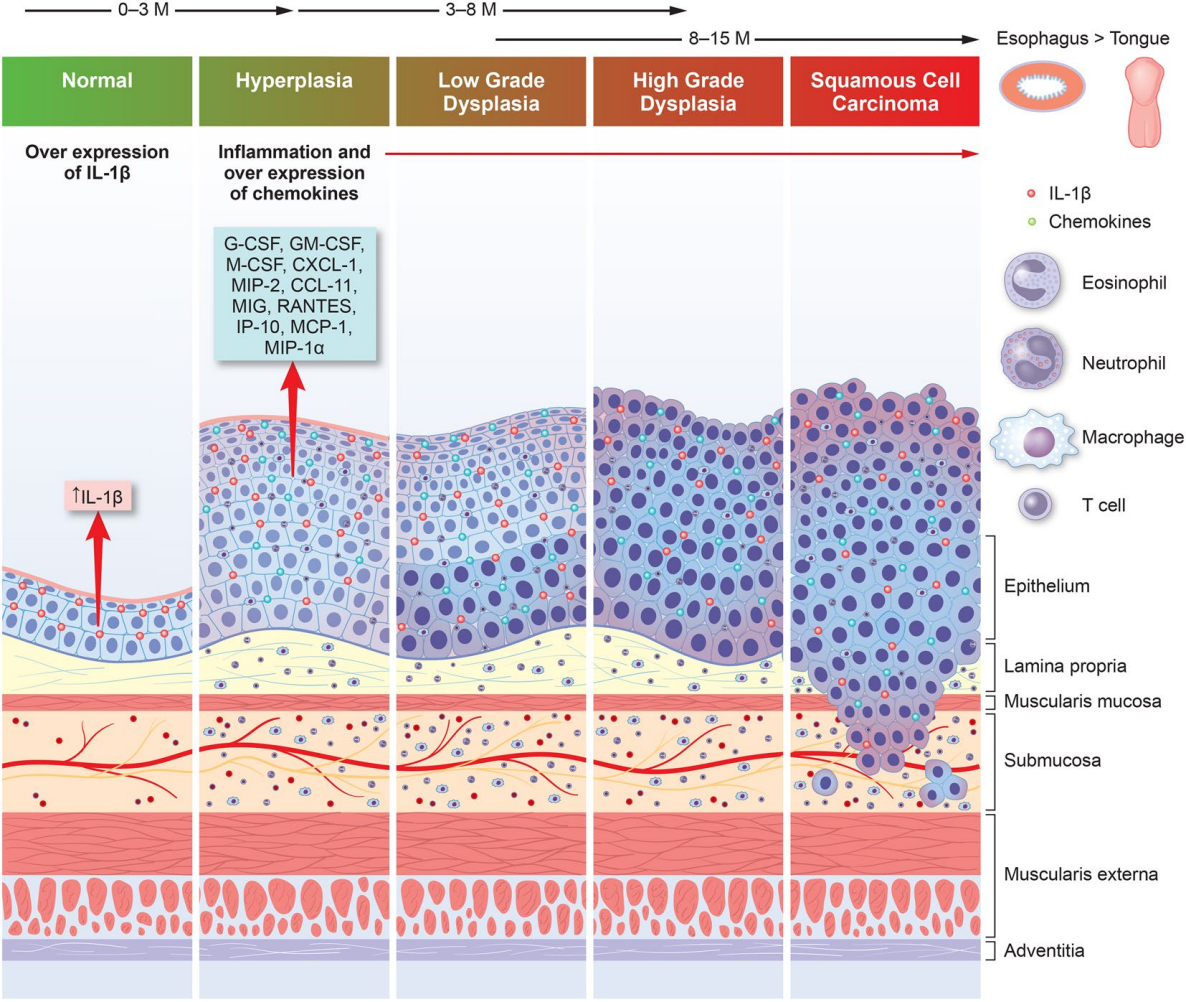
*IL-1*β mice as a model for Esophageal and Oral SCC progression: The graphical illustration depicts a cascade of events in *IL-1*β mice driven by IL1β overexpression leading to upregulation of inflammatory cells and cytokines with associated transformation of normal squamous epithelium to hyperplasia, dysplasia and esophageal and oral SCC (Graphical illustration—conceptualization, oversight and editing by S.M, D.A and JGF; created by Wendy Beth Jackelow, Medical and Scientific Illustration).

Chronic inflammation promotes secretion of a variety of proinflammatory and anti-inflammatory cytokines and diverse cellular factors with dynamic alterations in the phenotype of recruited cells and microenvironment during tumor promotion and associated poor prognosis in different tumors including esophageal cancers^47–49^. In human patients, gastric-esophageal reflux disease (GERD), Barrett’s esophagus (BE), esophageal squamous dysplasia and esophageal squamous cell carcinoma (ESCC) have been associated with increase in inflammatory cytokine stimuli such as IL-1β, IL-6, IL-8, IL-17 and TNFα^4,6,9,15,48^. In our *IL-1β* mouse model of ESCC, similar to previous observations in this model as noted by Munch et al.^27^ in the context of BE induced GEJ adenocarcinoma, the esophageal cytokines/chemokine profile in the tissue microenvironment is altered towards a more proinflammatory state. Various chemokines required for the recruitment of diverse types of inflammatory cells including T cells, granulocytes (neutrophils and eosinophils), macrophages and regulatory T cells were increased in the esophagus of the *IL-1β* mice during ESCC progression (Fig. 8). In human ESCC, cytokines and chemokines including TNFα^4^, IL-13^50^, VEGF^51^, LIF^52^, CCL11 and CXCL10^53^ are also enriched and correlate with poor clinical prognosis. Given these similarities, our *IL-1β* mouse model mimics the inflammatory landscape of human ESCC.

Tregs, specifically expressing Foxp3 + transcription factors, are considered major players that inhibit the function of immune effector cells with potential key roles in therapeutic strategies^14,33,34^. Treg abundance and Foxp3 + expression in esophageal cancer cells is correlated with a poor outcome in patients^37^ and increased Tregs in HNSCC also promotes evasion of host immunosurveillance mechanisms^33^. Further, IL-17A-producing subsets of CD4 + T cells accumulate in various tumors including esophageal cancer^35,48^. Immunosuppressive Myeloid Derived Suppressor Cells (MDSC’s) in oral tumors/SCC’s have been shown to secrete IL-6, IL-1β, IL-23 and PGE2 as well as promoting T-helper 17 (Th17) cell differentiation, which in turn activates expression of nitric oxide synthase (NOS) and cyclooxygenase 2 enzymes; all of which resulting in a sustained inflammatory milieu^49^. Interestingly, while IL-17A was significantly overexpressed in the *IL-1β* transgenic mice, it was undetectable in wild type littermates suggesting the unique role of IL-17A producing Th17 cells in the development of ESCC in our model. In addition, increased expression of IL-33 is associated with poor survival in esophageal cancer patients^38^. In the *IL-1β* model, Tregs, IL-17A and IL-33 are persistently elevated in the esophagus and tongue, and likely facilitate the progression of SCC.

Elevated tissue oxidative stress mediators such as reactive oxygen species (ROS) and reactive nitrogen species (RNS-iNOS and NO) in a chronic inflammatory state promote DNA double-strand breaks and genomic instability during the progression of esophageal squamous dysplasia and esophageal squamous cell carcinoma in human patients and mouse models^11,17–19^. SOX2 is an important biomarker with respect to squamous cell dysplasia/ carcinoma staging and progression, tumor invasiveness and patient prognosis in SCC’s^29,39,40^. Overexpression of IL-1β, specifically in the glandular murine stomach leads to severe inflammation and enhanced IL-6 and consequent activation of Stat3 and NF-κB pathways, and development of gastric adenocarcinoma^32^. SOX2 overexpression acting synergistically with Stat3 through inflammatory cytokines, IL-1β and IL-6, has been shown to cause malignant transformation of murine and human basal squamous epithelial cells and induce squamous tumors in the murine forestomach^29^.

In most rodent chemical carcinogenesis studies with 4-NQO, esophageal tumors/ESCC as well as oral squamous tumors were induced in both sexes by 24–66 weeks of age^20–22^. In a combined co-carcinogen mouse study utilizing 4-NQO and arecoline, two key carcinogens related to tobacco and betel quid respectively, it was shown that IL-1β is upregulated from secondary associated inflammation during the various stages of oral carcinogenesis^54^. Alcohol and cigarette smoking can also have synergistic, toxic effects on the promotion in humans by promoting genetic and epigenetic alterations including formation of DNA adducts and DNA damage^7^. Interestingly, oral 4-NQO treatment in mice does not necessarily lead to tumors/SCC development in the squamous fore stomach^55^, a feature similarly noted in the squamous stomach of our IL-1β mice. This lack of tumor promotion in the squamous stomach is puzzling as the *L2-IL-1β* mice are known to overexpress IL-1β in the squamous epithelium of esophagus and stomach^26^. We attribute the lack of dysplasia/SCC in the squamous stomach to one or more possibilities including higher resistance threshold of murine gastric squamous epithelium to injury from inherent adaptation to high gastric pH compared to the tubular esophagus, stem cell phenotypic differences based on anatomical location, and differences in microbiome and inflammatory mediators, all of which might have inhibited tumor promotion in the squamous stomach.

The *L2-IL-1β* mice described in our study is characterized by a robust inflammatory phenotype, a feature increasingly recognized to be a key player in in the prognosis of human head and neck, oral and esophageal SCC’s^33,36,38,41,42,45^. Of note, these studies have highlighted the role of granulocytes (eosinophils and neutrophils), T cells including regulatory T cells, macrophages and B cells within tumors and via circulation or in associated lymph nodes as predictors of disease prognosis and response to therapies. Interestingly, the inflammatory phenotype of esophageal SCC in *L2-IL-1β* mice is similar to that observed in the conditional KO model *p120 ctn* (catenin) of esophageal SCC, in which key factors such as GM-CSF, M-CSF, MCP-1 and TNFα are upregulated along with immature myeloid cell infiltration and desmoplasia within the tumor microenvironment^24^. In the *p120 ctn* conditional knockout mice, squamous dysplasia and neoplasia develop in the esophagus and squamous stomach in a comparable manner to our *L2-IL-1β* mice by 9–12 months of age. However, BE-type metaplasia or GEJ glandular dysplasia/adenocarcinoma was not documented in this model^24^. In the *ED-L2/Klf4* mice^25^, the progression of esophageal squamous dysplasia to overt invasive ESCC is much slower and ESCC are typically noted at around 2yrs, unlike our *L2-IL-1β* model in which ESCC is typically observed at 10–12 months of age. In comparison to the two well characterized GEM models of ESCC^24,25^, the *L2-IL-1β* mouse model offers the unique advantage of being a purely inflammation driven dual model of both ESCC and BE/GEJ adenocarcinoma development in the same animals.

While a sizable number of people develop exclusively either ESCC or BE, there are many reports of sporadic human cases of aggressive collision tumors with individuals simultaneously developing both ESCC and BE/Adenocarcinoma^56–59^. However, the molecular mechanisms by which both types of tumors develop in the same patients were not explored^56–59^. Given that our *L2-IL-1β* transgenic mice develop both BE/GEJ adenocarcinoma and ESCC, it provides the opportunity to further elucidate the differential molecular mechanisms driving these two distinct translationally relevant phenotypes that represent the two most common esophageal cancers in humans.

Based on epidemiological data and experimental studies, both oral and gutmicrobiome are thought to play a role in the carcinogenesis of gastrointestinal tract including oral and esophageal SCC’s^3,8,10,12^. Previous data from *1L-1β* mice maintained in a different institution documented attenuation of GEJ dysplasia and tumorigenesis in germ free (GF) conditions, and that this effect was augmented by a High Fat Diet (HFD) via fecal microbial alterations^27^. Likewise, in our study, the GEJ and gastric cardia/proximal corpus of *IL-1*β mice showed attenuated total histopathological index scores in the GF state as compared to SPF conditions, though dysplasia (columnar/glandular type) grades were similar in both conditions. Interestingly, in our study, *IL-1*β mice raised under both SPF and GF conditions showed similar grades/incidence of esophageal squamous dysplasia and ESCC development. Similarly, in a different study, 4-nitroquinoline treated GF and SPF mice showed comparable oral and esophageal tumorigenesis^23^.

In summary, we have shown that the L2*-IL-1β* mice are prone to spontaneously develop esophageal and oral SCC’s in addition the previously described phenotype of Barrett’s esophagus-like metaplasia^26,27^. In this model, IL-1β overexpression initiates a proinflammatory cascade with upregulation of various cytokines/chemokines and recruitment of inflammatory cells resulting in epithelial oxidative DNA damage, and progressive changes of squamous hyperplasia, dysplasia and SCC (Fig. 8). The *IL-1β* mouse model thus serves as a valuable tool of translational importance that in future studies could enable dissecting potential synergistic pathways during prolonged inflammation and carcinogen exposure in the multifactorial etiopathogenesis of BE and SCC in the esophagus and SCC of the oral cavity.

### Supplementary Information

Supplementary Information.

## Data Availability

All relevant experimental data are presented or summarized in the main narrative text and/ or supplemental material. Raw data files and additional images acquired as part of the study are available from the authors (SM, DA, ZG and JGF) upon request.
