# Supplementary material for: IL-1β transgenic mouse model of inflammation driven esophageal and oral squamous cell carcinoma
Source: Sci Rep. 2023 Aug 5;13:12732. doi: 10.1038/s41598-023-39907-8 (PMC10404242; doi:10.1038/s41598-023-39907-8)
Supplement: Supplementary file 1 — Supplementary Information. [file 41598_2023_39907_MOESM1_ESM.pdf]

Supplemental Methods

Histological grading scheme for GEJ and Glandular Stomach:

The gastric esophageal junction (GEJ), cardia and adjacent proximal corpus were scored as one segment (Supplemental Figure S3) due to difficulty in precisely delineating the different regions as a result of frequent coalescing pathological changes in *IL-1*β mice. The remainder 2/3rd of gastric corpus and antrum/pylorus as applicable were scored separately (data not presented) for inflammation, epithelial defects, oxyntic loss/atrophy, metaplasia (mucous type in GEJ and mucous /pseudopyloric metaplasia in gastric corpus), hyperplasia and dysplasia on a scale of 0(normal) to 4(severe change) as described earlier with slight modifications as needed for the different gastric compartments (27,30). For all categories, occasionally incremental scores of 0.5 were given when the lesions were ambiguous between two categorical scores. The sub-categorical scores were combined together to calculate Total pathology score per animal as shown in Supplemental Figure S3. The gastric antrum/pylorus was scored for inflammation, epithelial defects, hyperplasia and dysplasia.

Briefly, inflammation was evaluated on the basis of the amount and extent of leukocyte infiltration in the stomach as normal (score of 0), mild patchy focal mucosal and/or submucosal infiltration (score of 1), multifocal to coalescing mucosal and/or submucosal infiltration (score of 2), marked increased in leukocytes with lymphoid follicle formation+/- infiltration into the tunica muscularis (score of 3) and severe transmural multifocal to coalescing effacing inflammation (score of 4) . Epithelial defects were scored on the basis

of the extent of surface epithelial degeneration, tethering, erosions to full thickness ulcerations and associated glandular atrophy as absent (score of 0), rare (score of 1), frequent (score of 2), moderate with surface erosions with gland atrophy (score of 3) and severe with full thickness mucosal ulcerations, gland atrophy, and fibrosis (score of 4). Hyalinosis is a non- specific change characterized by the accumulation of brightly eosinophilic droplets and/or extracellular crystals and was scored on the basis of the extent of gastric mucosal involvement (30) as absent (score of 0), minimal with only surface intracellular epithelial glassy red hyaline material (score of 1), mild with presence of both intracellular hyaline material and/or occasional extracellular hyaline crystals, moderate with frequent extracellular crystals (score of 3), and severe with diffuse hyaline change/crystals in the scored segment (score of 4). Oxyntic atrophy was defined as a reduction in the mass of chief and parietal cells in the gastric corpus segments and graded on the extent of loss via cell loss and/or metaplastic transformation. Oxyntic atrophy in the different corpus segments were scored as no oxyntic loss/atrophy (score of 0), minimal involving loss/atrophy of less than 25% of oxyntic cells (score of 1), mild with loss of approximately 26-50% of oxyntic cells (score of 2), moderate involving loss/atrophy of approximately 51-75% of oxyntic cells (score 3), and severe with more than 75% to complete loss of oxyntic cells, parietal and chief cells (score of 4).

Epithelial hyperplasia was scored on the basis of increased gastric mucosal thickness by virtue of proliferation of surface foveolar-type epithelium and/or antral-type glandular units and appropriately graded in relation to the extent of oxyntic loss. Epithelial hyperplasia was scored on the basis of increased length/depth of foveolar lining epithelium and/or glandular size/length as normal (score of 0), minimal (~1.5 times normal length/size, score of 1), mild (~2 times normal length/size, score of 2), moderate (~3 times normal

length/size, score of 3), and severe (≥ 4 times normal length/size). Mucous metaplasia reflects a morphological change in appearance of glandular epithelium resulting from expansion of gastric mucous neck cells (PAS+/Alcian blue/TFF2 +) with foamy cytoplasm and marginated nucleus resembling Brunner's glands and usually secreting a mixture of neutral and acidic mucins in the gastric corpus mucosa. Pseudopyloric metaplasia is a preneoplastic change defined as replacement of the oxyntic mucosa by glands resembling antral phenotype with cells being more columnar and lacking typical cytoplasmic granules of oxyntic cells as well as the absence of cytoplasmic mucous appearance of mucous metaplasia. Both mucous metaplasia and pseudopyloric metaplasia were scored in the different corpus compartments on the basis of extent of oxyntic cell transformation as none (score of 0), minimal (rarely present, score of 1), mild with involvement of less than 1/3rd of corpus segment (score of 2), moderate with involvement of 1/3rd to 2/3rd of corpus segment (score of 3), and severe with involvement of more than 2/3rd of scored corpus segment.

Gastric epithelial dysplasia is histologically an unequivocal neoplastic change without evidence of stromal invasion occurring in sessile, flat, depressed or elevated/polypoid mucosal lesions and is defined by both architectural abnormalities (haphazard glandular arrangement, loss of vertical orientation, back-to-back gland associations, branching, infoldings, and piling up of cells) and cytological atypia (cellular pleomorphism, anisocytosis, anisokaryosis, ill-defined cellular junctions, loss of nuclear polarity, pencil or cigar shaped nuclei, hyperchromatic nuclei, increased nuclear–cytoplasmic (N–C) ratio, visible mitosis, bizarre mitotic figures) (30). The term gastric adenoma was restricted to well demarcated or circumscribed, polypoid or raised nodular lesions comprising of tubular and/or villous structures, lined by dysplastic epithelium.

Dysplasia/neoplasia was graded on its extent and severity of changes as follows: score of 0 for normal or mild simple epithelial hyperplasia with no atypia; score of 1 for one or few epithelial hyperplastic foci with mild architectural atypia (equivalent to indefinite for dysplasia or reactive epithelium); a score of 2 for atypical hyperplasia comprising of coalescing proliferative epithelial lesions with glandular architectural abnormalities (equivalent to indefinite for dysplasia); a score of 2.5 for low grade dysplasia/intraepithelial neoplasia or low grade adenoma and all of these proliferative lesions were defined by severe glandular architectural abnormalities and borderline cytological atypia; a score of 3 is equivalent to high grade dysplasia/intraepithelial neoplasia or a high grade adenoma characterized by severe architectural and cytological atypia; a score of 3.5 for intramucosal invasive neoplasia (intramucosal carcinoma) characterized by high grade dysplastic lesions with unequivocal invasion into the lamina propria or muscularis mucosa (dysplasia score of 3.5; and a score of 4 for submucosal invasive neoplasia (submucosal carcinoma) for true unequivocal invasion into the gastric submucosa or beyond.

Supplemental Table 1

Mice / Age	SPF WT (C57BL/6)		GF WT (C57BL/6)		SPF L2- *IL-1β*		GF L2- *IL-1β*	
	m	f	m	f	m	f	m	f
3M	2	1	-	-	1	2	-	-
6M	9	5	-	-	5	5	-	-
8M	6	7	-	-	4	5	-	-
10M	6	6	-	-	6	5	-	-
12-15M	9	8	3	4	8	10	12	10
12-13M GF born-SPF	-	-	-	-	3	6	-	-

SPF= Specific Pathogen Free; GF= Germ Free; WT= wild type;
M= months; m= male; f= female

Supplemental Table 1: Tabular numerical data on the various experimental mice, groups, age/sex and housing conditions

Supplemental Figure S1

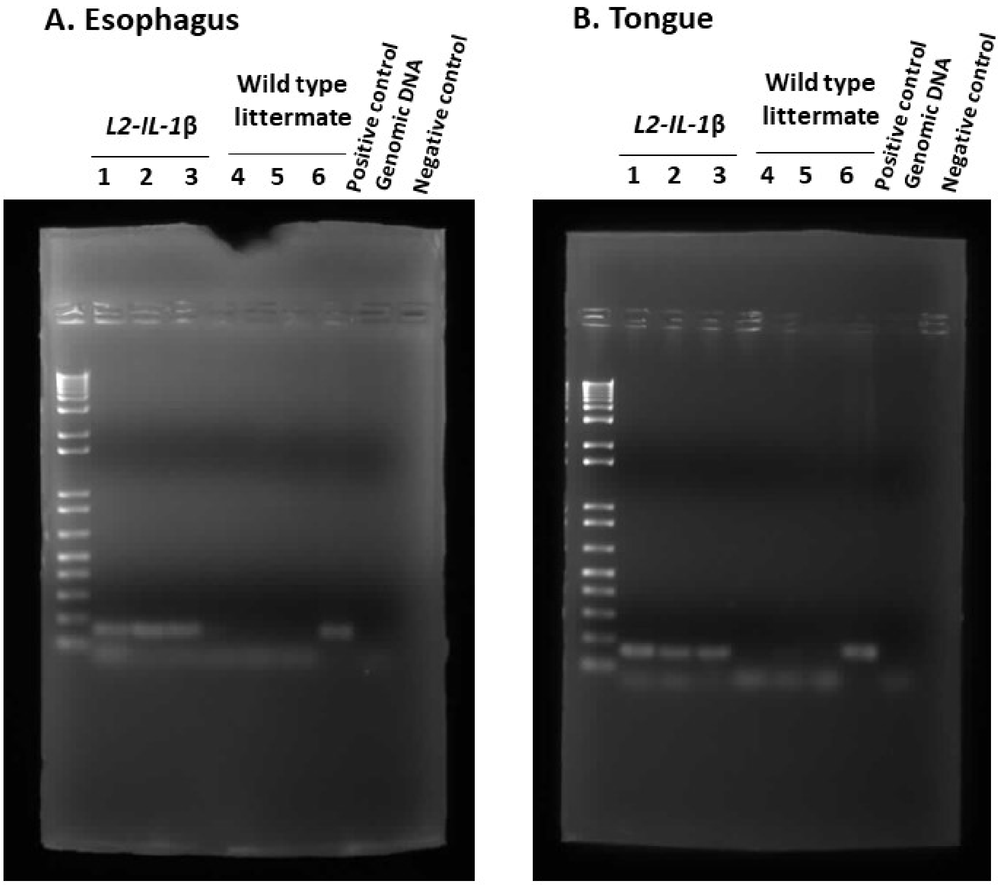

Supplemental Figure S1: Over expression of human IL-1β in *L2-IL-1*β transgenic mice. Representative gel panel image depicting mRNA level of hIL-1β in (A) esophageal and (B) tongue tissues was determined by semi-quantitative PCR. WT mice (n=3, SPF), *IL-1*β mice (n=3, SPF).

Supplemental Figure S2

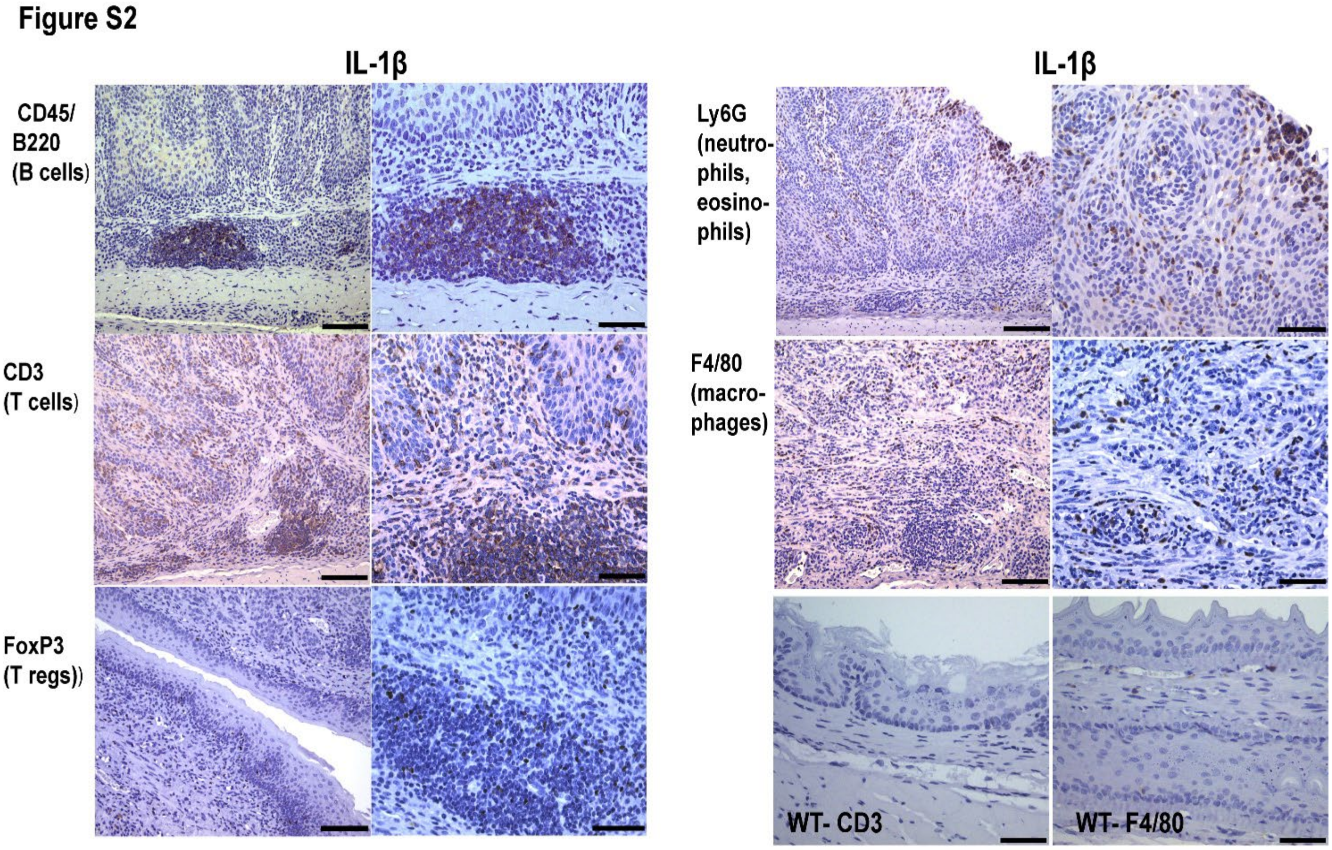

Supplemental Figure S2: Representative immunohistochemical images of the esophagus of *IL-1β* for various immune cell markers; Left panel- CD45/B220 (B cells), CD3 (T cells), FoxP3 (T regs), low and high magnification. Right panel (top 4 images)- Ly6G (granulocytes- neutrophils and eosinophils), F4/80 (macrophages), low and high magnification. Representative high magnification images of WT control esophagus for CD3 and F4/80. WT (n= 5, 3 GF and 2 SPF) and IL-1β mice (n=8, 4 GF and 4 SPF). Scale Bars: IL-1β, left panels- 200μM, right panels- 100μM; WT, Right bottom two images- 100μM.

Supplemental Figure S3

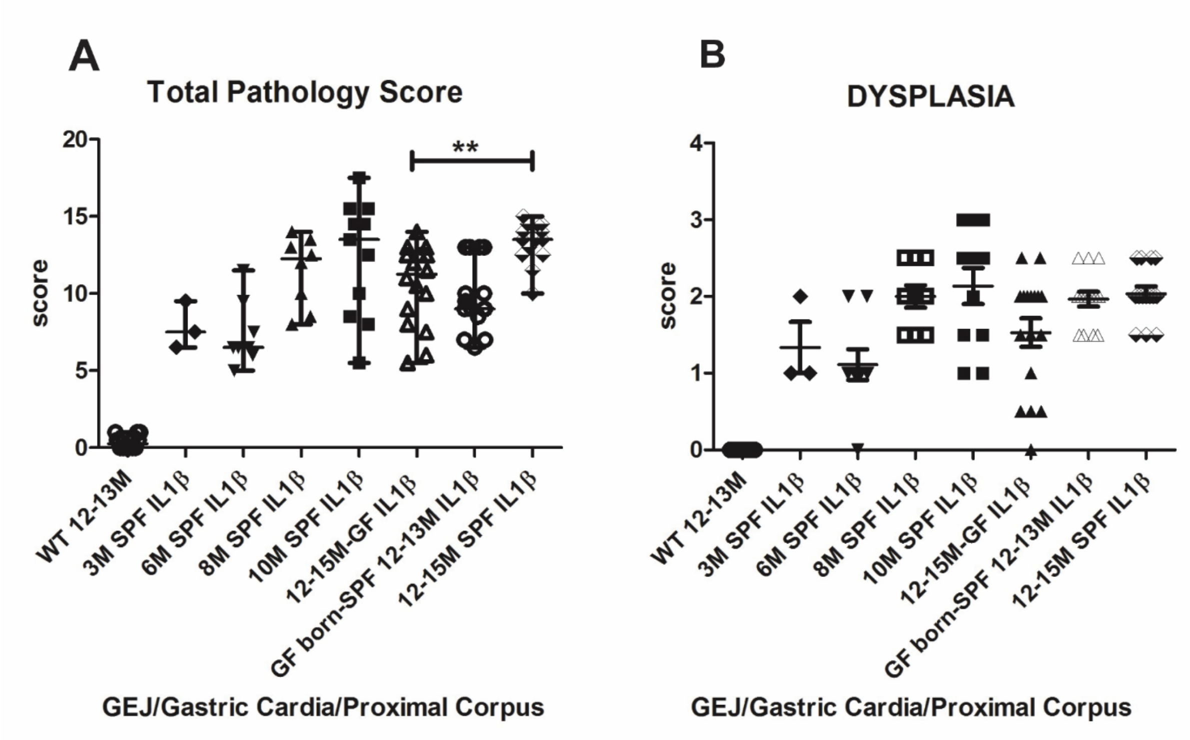

Supplemental Figure S3: Histopathological scoring of the gastric-esophageal junction (GEJ) and gastric cardia/proximal corpus of wild type (WT) and *IL-1β* transgenic mice: A. Cumulative histopathology index score bar chart for different age groups (M-months) of mice raised in different conditions; GF- germ free, SPF- specific pathogen free, GF born - SPF (transferred at 3-4 months of age). Histopathological criteria included inflammation, epithelial defects, oxyntic loss/atrophy, metaplasia (mucous type), epithelial hyperplasia (foveolar and glandular) and dysplasia/invasive neoplasm (columnar type, surface and glandular) graded on a scale of 0 to 4. B. Scatter/dot plot of dysplasia scores of different groups of mice. Individual n numbers per group/timepoint are listed in Supplemental Table 1. P values where significant and relevant are denoted by *.

Supplemental Figure S4

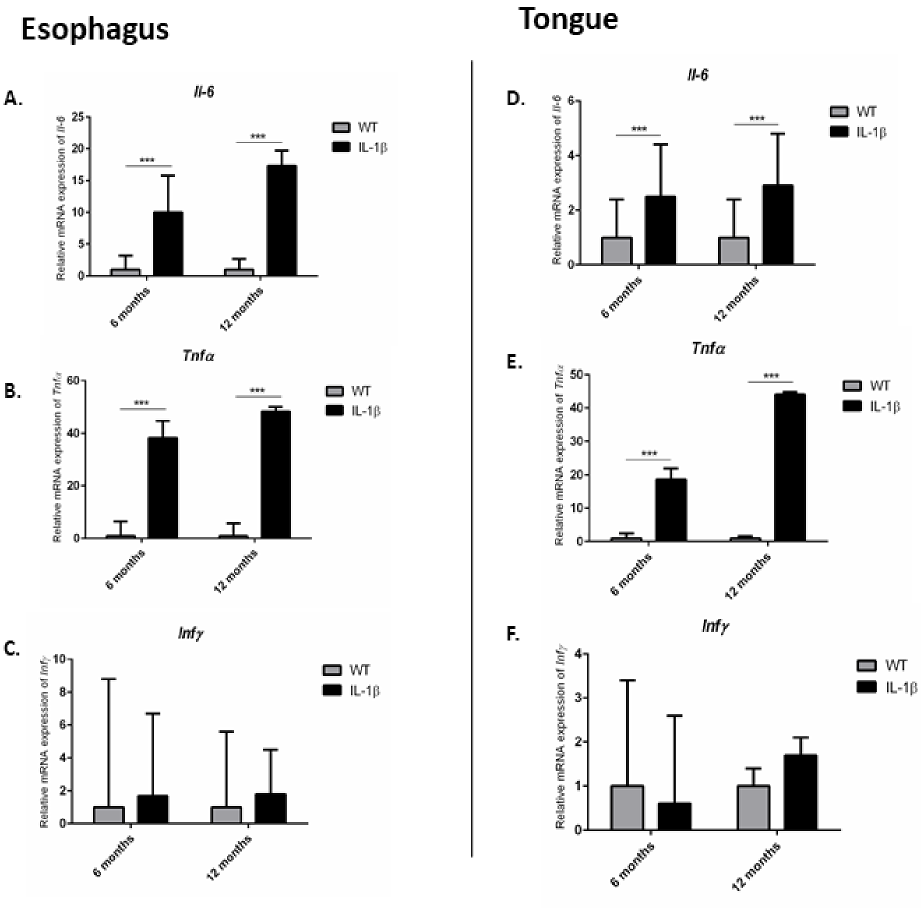

Supplemental Figure S4: Upregulation of pro-inflammatory cytokines downstream of IL-1β signaling in esophagus and tongue of *IL-1β* transgenic SPF mice. Relative mRNA level of genes contributing to inflammation were determined in the esophageal (A-C) and tongue (D-F) tissues of 6M and 12M *IL-1β* SPF mice. All the mRNA levels were normalized to the expression of housekeeping gene *Gapdh*. (A), *Il-6* .(B) *Tnfα* (C) *Infγ* were determined in the esophageal tissues and (D) *Il-6,* (E) *Tnfα* (F) *Infγ* were determined in the tongue tissues. The *y*-axes represent the mean fold changes (± standard deviation) of the mRNA levels in reference to wild type littermates. ***$p \leq 0.001$. n= 10 -13/group/tissue.

Supplemental Figure S5

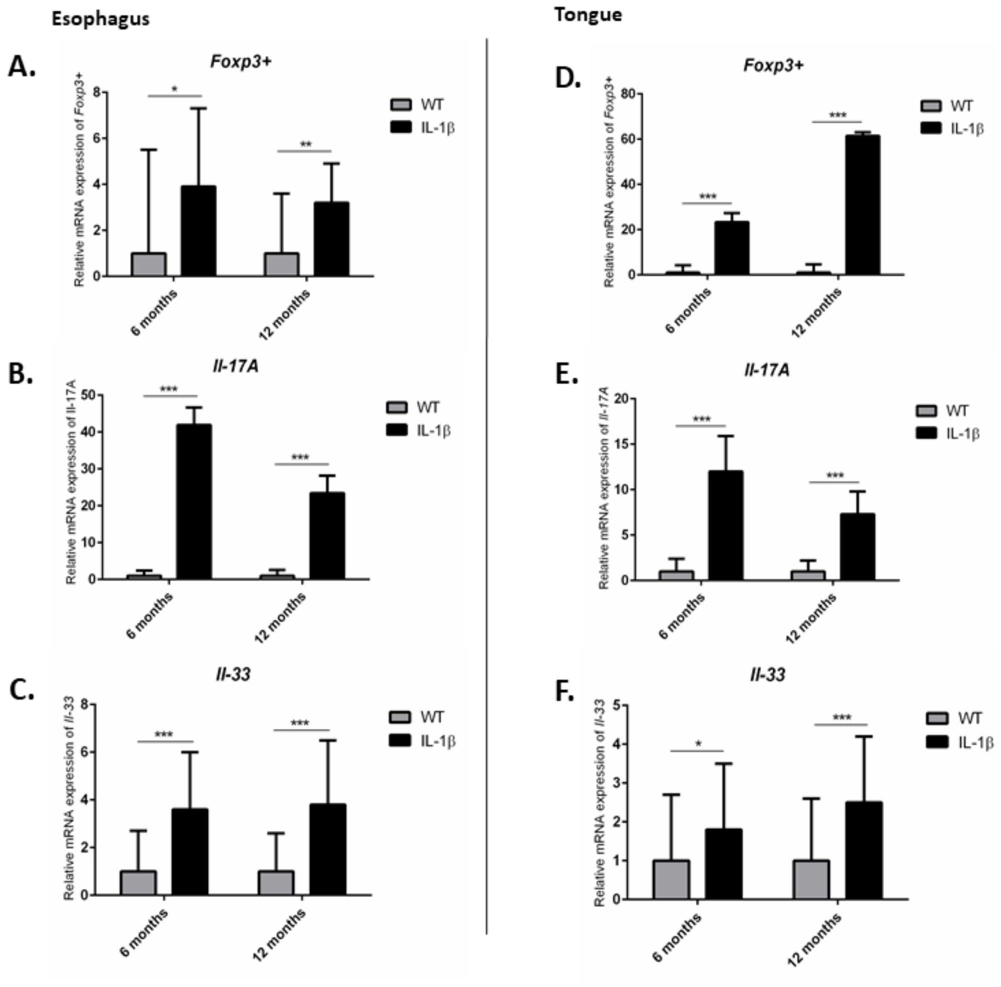

Supplemental Figure S5: Upregulation of *Foxp3+*, *Il-17A* and *Il-33* in esophagus and tongue of *IL-1β* transgenic SPF mice. Relative mRNA level of *Foxp3*+, *Il-17α* and Il-33 were determined in the esophageal (A-C) and tongue (D-E) tissues. All the mRNA levels were normalized to the expression of housekeeping gene *Gapdh*. (A) *Foxp3*+ (B) *Il-17A* (C) *Il-33* were determined in the esophageal tissues and (D) *Foxp3*+ (E) *Il-17A* (F) *Il-33* were determined in the tongue tissues. The *y*-axes represent the mean fold changes (± standard deviation) of the mRNA levels in reference to wild type littermates. *p≤0.05, **p≤0.01, ***p≤0.001. n= 10 -13/group/tissue.
